# A design of experiments screen reveals that *Clostridium novyi*-NT spore germinant sensing is stereoflexible for valine and its analogs

**DOI:** 10.1038/s42003-023-04496-9

**Published:** 2023-01-28

**Authors:** Ajitha Sundaresan, Mai Le Ngoc, Marvell Ung Wew, Varsha Ramkumar, Prahlad Raninga, Rongji Sum, Ian Cheong

**Affiliations:** 1 Temasek Life Sciences Laboratory, Singapore, Singapore; 2 Department of Biological Sciences, National University of Singapore, Singapore, Singapore; 3 NUS High School of Mathematics and Sciences, Singapore, Singapore

**Keywords:** Bacterial development, Bacteriology, Bacterial infection

## Abstract

Although *Clostridium novyi-*NT is an anti-cancer bacterial therapeutic which germinates within hypoxic tumors to kill cancer cells, the actual germination triggers for *C. novyi*-NT are still unknown. In this study, we screen candidate germinants using combinatorial experimental designs and discover by serendipity that D-valine is a potent germinant, inducing 50% spore germination at 4.2 mM concentration. Further investigation revealed that five D-valine analogs are also germinants and four of these analogs are enantiomeric pairs. This stereoflexible effect of L- and D-amino acids shows that spore germination is a complex process where enantiomeric interactions can be confounders. This study also identifies L-cysteine as a germinant, and hypoxanthine and inosine as co-germinants. Several other amino acids promote (L-valine, L-histidine, L-threonine and L-alanine) or inhibit (L-arginine, L-glycine, L-lysine, L-tryptophan) germination in an interaction-dependent manner. D-alanine inhibits all germination, even in complex growth media. This work lays the foundation for improving the germination efficacy of *C. novyi*-NT spores in tumors.

## Abstract (web summary)

A screen for germination of *Clostridium novyi*-NT spores reveals that germinant sensing of this tumor-destructive anaerobic bacterium is stereoflexible for valine and its analogs and identifies co-germinants to improve germination efficacy in tumors.

## Introduction

*Clostridium novyi* Type A is an obligate anaerobic spore-forming bacterium which causes clostridial myonecrosis, big head, and necrotic hepatitis in sheep, goats and swine^1^. Notably, it is responsible for ‘Dikkop’ Swollen Head in ram, an infection which occurs through wounds inflicted between young rams when fighting, with death occurring within 48–72 h^2^. *C. novyi* is more a pathogen in cattle than in humans, with the exception of a sporadic outbreak of infections among drug-users in Scotland whose heroin had been contaminated by *C. novyi* spores^3^.

*C. novyi*-NT, a non-lethal version of the wildtype bacteria generated through attenuation of its alpha-toxin, is currently in clinical testing as an experimental therapeutic for solid tumors^4,5^. *C. novyi*-NT’s therapeutic potential derives from its ability to selectively colonize the hypoxic regions (<0.5% oxygen) of tumors and to exert a localized cytotoxic effect^6^. Although other clostridia prior to *C. novyi*-NT have been investigated for their tumor-killing effect, *C. novyi-*NT was the first to be administered as spores instead of vegetative rods^4^. Besides being relatively non-immunogenic, administering *C. novyi*-NT as spores adds an extra layer of tumor-specificity because germination is favored within the hypoxic tumor microenvironment^4,6^. Spores, being dormant, are also resistant to the oxygen which would otherwise kill them in their vegetative form^6^. Although spores are hence the ideal clostridial form for tumor therapy, little is known about what makes a *C. novyi*-NT spore germinate, other than the requirement for a reducing environment. Further, what makes *C. novyi*-NT an effective colonizer for some tumors but not for others remains a mystery^7^.

Our current knowledge of bacterial spores and germination are largely based on the Bacillus and Clostridium genera^8,9^. In essence, bacteria sporulate when metabolic resources are scarce but germinate when resource-rich conditions again prevail. The ability to patiently await the return of favorable conditions in a dormant state encapsulates the survival advantage of sporulating bacteria. In pathogenic bacteria, this cyclic process drives pathogenesis, as once benign spores infect their target environments, germinating into their toxin-producing vegetative forms^10–12^. Germination can be divided into two stages^13^. The first stage begins with the interaction of small molecule nutrient germinants with specific proteins called germination receptors (GRs). GR activation causes the release of monovalent cations (H^+^, K^+^, and Na^+^) and calcium dipicolinate (CaDPA), as well as the partial hydration of the spore core. In the second stage, the spore cortex layer is hydrolyzed, leading to core expansion and complete hydration, completing the transition from dormancy. Subsequently, DNA, protein synthesis, and key metabolic pathways become active, resulting in the outgrowth of the vegetative form^14^.

Bacterial spores are known to germinate in response to a wide range of factors such as amino acids^15^, purines^16^, sugars^17^, bile salts^18^, and ions^19^. Germinants are important correlates of nutritive environments, and sporulating bacteria have thus evolved to use them as signals to exit spore dormancy. Screening for germinants is typically performed by detecting spore germination, signaled by a drop in optical density or refractility, in the presence of single candidate germinants. However, actual germination environments are complex and contain multiple factors which can potentiate or antagonize the effect of any putative germinant. For example, *Clostridioides difficile* spores germinate in the presence of the bile salt taurocholate. In the gut environment, however, this germination can be antagonized by other microbe-derived secondary bile salts such as lithocholate and ursodeoxycholate^20^. In the opposite scenario, putative germinants requiring positive interactions with other factors in the environment might yield a false negative result when evaluated as a single candidate.

Screening candidate germinants singly one-by-one will always give an incomplete picture if interactions among them exist. An approach that screens for germination factors in combinations is therefore needed because the traditional intuition to change only one factor at a time becomes exponentially inefficient as the number of variables increases. In a Design of Experiments (DOE) approach, multiple factors are changed simultaneously in a structured manner^21^. In the realm of spore biology, DOE has been used to optimize media components for bacterial sporulation^22–24^ and cultivation^25–27^. In this study, we use DOE to identify germinant factors with the most significant effects, as well as interactions between them. These experiments revealed germinant and co-germinant candidates, and further led to the serendipitous observation of D-valine and its analogs as potent germinants of *C. novyi-*NT.

## Results

### How DOE was used in this study

Supplementary Table 1 is an example of a DOE design, where rows represent independent experiments and columns represent tested factors. Each row tests a different combination of tested factors which are either present (+) or absent (−). Each column has an equal number of + and − levels, and any two randomly chosen columns placed adjacently have an equal number of paired levels (++, +−, −+, and −−). Hence, each level (+ or −) of each factor associates an equal number of times with each level (+ or −) of every one of the other factors. The + and − levels of any factor can thus be directly compared with each other since both are subject to exactly the same balanced background from the other factors. Used in this way, DOE screens estimate the most significant factors among a host of candidate factors using an efficient number of experimental runs.

**Fig. 1:**
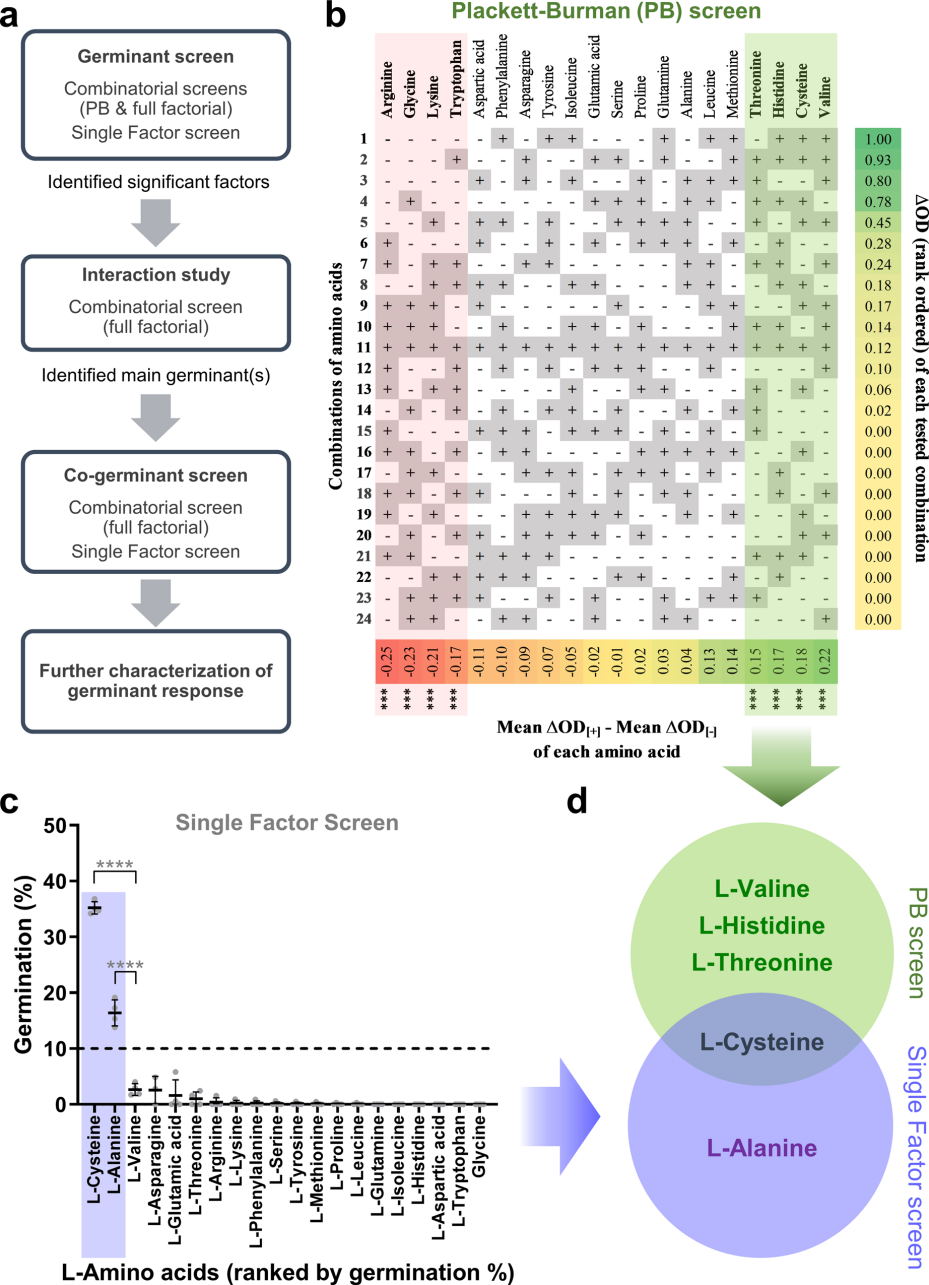
Plackett–Burman and single factor screens for L-amino acid germinants. **a** Schematic of the Design of Experiments (DOE) approach. **b** 20 L-amino acids at concentrations in Supplementary Table 3 were added in different combinations according to the Plackett-Burman (PB) design in Supplementary Table 1. Figure depicts the different combinations sorted in decreasing order (from top to bottom) of germination response expressed as ΔOD (extreme right column) as defined in Supplementary Table 4. Figure also shows the effect (bottom row) of each L-amino acid on the ΔOD, arranged in increasing order from left to right. Factors highlighted in red and green at the two extreme ends of the matrix represent the L-amino acids with the most significant antagonist and pro-germination effects respectively (ɑ = 0.0005). All data were averaged from two independent experiments with two replicates each and represent mean response values. **c** 20 L-amino acids at twice the concentrations in Supplementary Table 3 were added individually per well. The germination extent scatter dot plot for 20 L-amino acids as single factors is shown (mean ± SD), *n* = 4 biologically independent samples. Dashed line represents the 10% threshold for germination. L-amino acids are sorted according to their germination % (as defined in Supplementary Table 4), with highest to lowest from left to right. Shaded area in purple highlights L-amino acids which show significant germination compared to L-valine (Unpaired t-test, ****p < 0.0001). **d** Venn diagram of significant pro-germination candidates discovered through the PB screen (green) and single factor screen (purple).

We characterized the germination requirements for *C. novyi-*NT in three ways, screening for primary germinants, co-germinants, and interactions among identified candidates (Fig. 1a). The Plackett-Burman (PB) design (Supplementary Table 1) was used in initial experiments where the number of factors to be tested was high (>5). Once this number was filtered to the key pro-germinants, a full factorial design (Supplementary Table 2) was then used to analyze any interactions amongst them. In all experiments, germination was measured by the drop in OD600 over time. All germination positive readings were verified by loss of refractility under phase contrast microscopy. High non-physiological concentrations were first used in the germinant screens to minimize false negatives. Dose response studies were subsequently used to study the screened germinants at physiological concentrations. In this study, all germinant candidates are cataloged in Supplementary Table 3, technical terms used in the DOE graphs are defined in Supplementary Table 4, and all statistical tests performed are listed in Supplementary Table 5.

### DOE and single factor screens identify the key L-Amino acid germinants

L-amino acids signal the presence of nutrients and are the most common germinants for bacterial spores. We took the 20 canonical L-amino acids (Supplementary Table 3) and tested them for germinant properties using two parallel approaches. In the first, we tested combinations of L-amino acids using a specific DOE approach called the Plackett-Burman (PB) design^28^. In the second, we tested each L-amino acid independently as is usually done.

Why perform PB alongside a regular germinant screen? First, a PB screen would identify not just germinants but also germination antagonists. The second reason has to do with interactions. PB, which by design is based on combinations of factors, will yield discrepant results with the single factor screen if interactions exist between factors. This is a limitation of PB when it is used for the primary goal of screening for independent factors. Here, we intentionally use this property to reveal the existence of interactions between the L-amino acids. After all, it is conceivable that interactions might exist to render a germinant L-amino acid more or less potent in the presence of another L-amino acid. Conversely, if interactions did not exist, both screens should yield identical results.

For the PB screen, 24 combinations of the 20 canonical L-amino acids were constructed so that each amino acid was present in half the combinations and absent in the other half (Supplementary Table 1). A PB design requires just a fraction of all possible 2^20^ permutations of L-amino acids to be tested, allowing a single PB experiment with kinetic OD600 measurements to be performed in a single microtiter plate with each well corresponding to one combination. Two PB experiments (each with two replicates per combination) were performed, and the normal plot of standardized effects is shown in Supplementary Fig. S1. Additionally, a more intuitive data representation of the ΔOD for each combination and the effect associated with each L-amino acid is shown in Fig. 1b. The columns show candidate germinants rank ordered by their associated effect on germination, whereas the rows show combinations of these candidates rank ordered by germination response. In this study, we use the term ‘pro-germinant’ or ‘pro-germination factor’ to refer to a factor which promotes germination, regardless of whether it acts independently (e.g., as a primary germinant) or in combination (e.g., as a co-germinant). Along the columns, four pro-germination factors (L-valine, L-cysteine, L-histidine and L-threonine), as well as four anti-germination factors (L-arginine, L-glycine, L-lysine and L-tryptophan), were identified as having significant effects (99.95 CI, model fit *R*^2^ = 92.1%). Since the rows represent combinations and these are similarly rank ordered by germination response, one would expect if there were no interactions, that the row containing every single candidate germinant would be ranked at or near the top. Instead, we see that the row containing all candidates is in row 11 out of 24. Using this row as a visual reference, the pro-germination factors are enriched above row 11 to the top right. Conversely, the anti-germination factors are enriched below row 11 to the bottom left. This squares with our intuition that subtracting the anti-germinant factors from row 11 should increase the germination response, and these combinations should be ranked above row 11. On the other hand, subtracting pro-germination factors from row 11 would have the opposite effect by decreasing the germination response, and these combinations would be ranked below row 11. These results show that we miss important information by not taking interactions into consideration. In parallel with the PB screen, a single factor screen was performed with each of the 20 L-amino acids independently evaluated as germinant candidates (Fig. 1c). Here, L-cysteine and L-alanine significantly induced greater than 10% germination and were identified as germinants.

Taken together, both the PB and single factor results show that interactions do indeed modulate the activity of germination factors in ways that are not reflected by a typical single factor germination screen (Fig. 1d). L-cysteine was the only germinant identified in both screens, showing that its germinant abilities were effective even when admixed with other amino acids. The remaining germinants were identified either in the PB or single factor screen alone. L-alanine, for example, was identified only in the single-factor screen, suggesting that its germinant abilities as a single agent were masked by inhibitory interactions with other amino acids. Finally, L-valine, L-histidine, and L-threonine were identified as germinants in the PB screen alone. This shows that certain L-amino acids, while inert by themselves, can likely promote germination in the presence of other factors in the environment.

### Germination by L-cysteine is highly modulated by inhibitory interactions

**Fig. 2:**
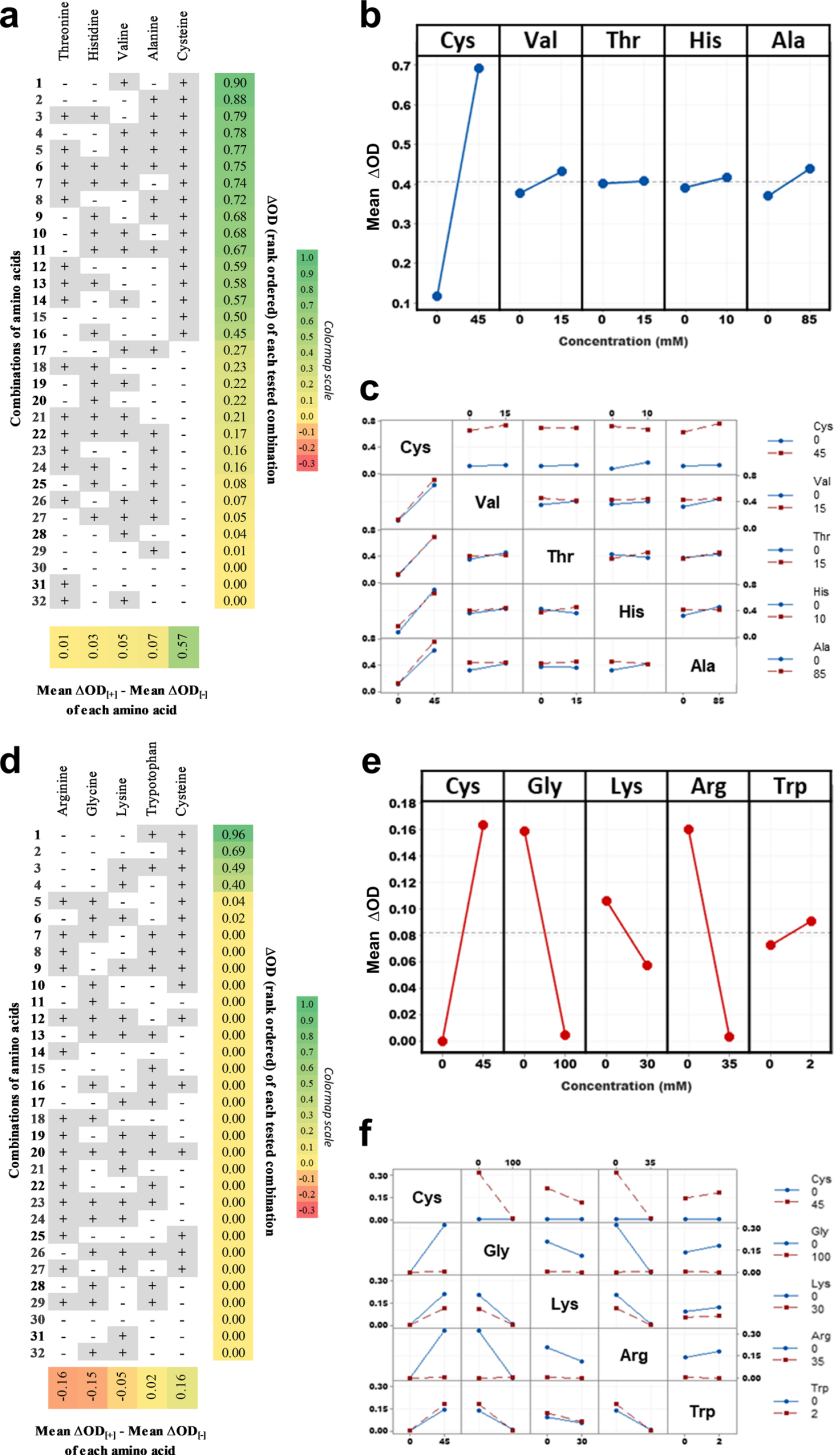
Germinant interaction study of L-amino acid hits from PB and single factor screens. Full factorial experiments were performed involving the pro-germination factors (panels **a**–**c**) and antagonists of L-cysteine germination (panels **d**–**f**). Both experimental designs are detailed in Supplementary Table 2. Concentrations used were the same as the Plackett-Burman screen. **a**, **d** Figures depict the different combinations of factors, sorted in decreasing order (from top to bottom) of ΔOD (extreme right column) as defined in Supplementary Table 4. The effect (bottom row) of each L-amino acid on ΔOD, arranged in increasing order from left to right is also shown. **b**, **e** Main interaction plot of pro-germination L-amino acids (blue lines with circles, model ɑ = 0.0001) and antagonist L-amino acids with L-cysteine (red lines with circles, model ɑ = 0.001) respectively showing the average of ΔOD for each amino acid concentration. **c**, **f** Secondary interaction plot showing the interactions between all pairs of factors. Blue lines with circles represent absence, and red lines with circles represent the presence of indicated L-amino acids. All data were averaged from two independent experiments with two replicates each and represent mean response values. Positive slopes indicate positive effects on germination, while negative slopes indicate inhibitory effects.

Next we studied the interactions among the 5 L-amino acids (Fig. 1d) identified as having a positive effect on germination by testing all 2^5^ combinations of these germinants for interactions between them (Fig. 2a and Supplementary Table 2). Similar to Fig. 1b, the columns in Fig. 2a show the pro-germinants ranked by their effect on germination and the rows show combinations of these pro-germinants ranked by germination response. In these results, L-cysteine occupies the top ranked rows and is absent from the bottom rows. In short, a high germination response is achieved only if L-cysteine is present. L-cysteine’s dominance as a pro-germinant relative to the other L-amino acid pro-germinants is also reflected in the alternative visualization in Fig. 2b, which shows the germination response as a slope instead of a heatmap. L-cysteine’s dominance coincides with it being the only amino acid identified in both the PB screen and single factor screen. This perhaps reflects that L-cysteine has a strong primary germinant effect which is not easily antagonized by the other L-amino acids. L-valine and L-alanine in contrast, were weaker runners-up (Fig. 2a, b). Digging deeper, we asked if there were any significant interactions between the 5 L-amino-acid pro-germinants. The results from this analysis are shown as a secondary interaction plot, which shows the germination response for every possible amino acid pair when either is present or absent (Fig. 2c). Each boxed graph sits at the intersection of two amino acid labels, one along the same row as the box (the “row factor”), and the other along the same column as the box (the “column factor”). Within each box graph, the blue line establishes the baseline germination response for the column factor and the red line shows the modified germination response when the row factor is present. If there are no interactions, then the expected result is that the red and blue lines in each box graph will be approximately parallel. Since this is the observation in Fig. 2c, the conclusion is that there are no strong interactions between the 5 L-amino acid pro-germinants.

Since the PB screen had also identified amino acids which negated germination, we used the same set of full-factorial analyses to study the effect of these 4 negative factors (glycine, L-arginine, L-lysine, and L-tryptophan) on L-cysteine induced germination. In the full-factorial heatmap, it can be seen that L-cysteine is necessary but insufficient for a strong germination response (Fig. 2d). L-cysteine is present in the top 4 rows where the only significant germination occurs. L-cysteine’s germinant effect drops to zero when either L-arginine or L-glycine are present. The alternative visualization in Fig. 2e, confirms this observation, and further reveals a weaker interaction with L-lysine. This intuition is further reinforced by the secondary interaction plot (Fig. 2f), which shows non-parallel blue and red lines for multiple box graphs. As expected, a deviation from parallel is observed for the 4 box graphs related to L-cysteine + L-glycine and L-cysteine + L-arginine. Additionally, there are also deviations from parallel for the 2 box graphs related to L-arginine and L-glycine. In both these graphs, the absence of both L-arginine and L-glycine is associated with high germination triggered by L-cysteine. The presence, however, of either L-arginine or L-glycine or both, results in no germination. The above full factorial analyses imply that inhibitory amino acids in the natural environment may act as antagonistic modifiers to L-cysteine’s strong pro-germinant effect. Interestingly, L-tryptophan, which had an inhibitory effect in the PB screen showed a very slightly positive effect on germination. The effect of certain amino acids on germination may hence be highly contextual, emphasizing the need to probe the combinatorial space of candidate germinants.

### Hypoxanthine is a potent co-germinant for L-cysteine-mediated germination

**Fig. 3:**
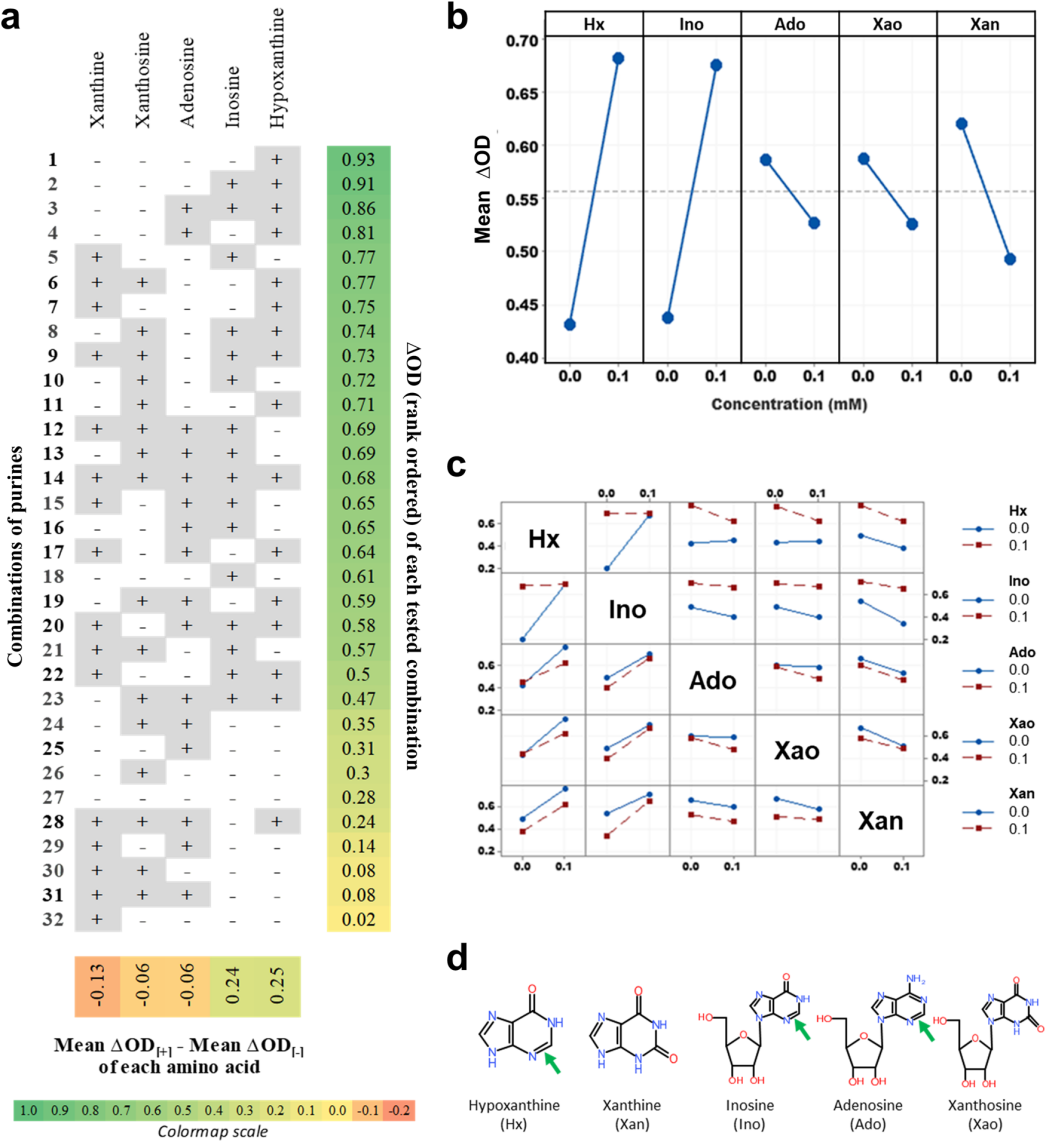
Purine co-germinant full factorial screen with L-cysteine. Germination response was measured in the presence of L-cysteine (10 mM) for all combinations of purines (0.1 mM) according to the factorial design detailed in Supplementary Table 2 (panels **a**–**c**). **a** Figure shows the different combinations of purines in the presence of main germinant L-cysteine, sorted in decreasing order (from top to bottom) of ΔOD (extreme right column) as defined in Supplementary Table 4. Figure also shows the effect (bottom row) of each purine on ΔOD, arranged in increasing order from left to right. **b** Main interactions plot for the five purine analogs (blue lines with circles, model ɑ = 0.0005). **c** Secondary interaction plot showing the interactions between all pairs of factors. Blue lines with circles represent absence and red lines with circles represent presence of indicated purines. **d** Structures of the five purine analogs. A reduced purine ring featuring a C2-N3 double bond (green arrow) is associated with germination. All data were averaged from two independent experiments with two replicates each and represent mean response values.

We next tested purine derivatives, which are the next most common germinant class for bacterial spores after L-amino acids. Purine candidates were tested in parallel using the full factorial and single factor screens but none were found to trigger germination in either screen (Supplementary Figs. S2a–S2c). Since purines have been known to act as co-germinants^29^, and the number of purines tested was low, we designed a full factorial screen with all possible combinations of 5 purines. A sub-optimal L-cysteine concentration insufficient to trigger full germination was used as the baseline condition. Any co-germinant activity to L-cysteine would hence be revealed by an increased drop in OD600. The results demonstrate unequivocally that hypoxanthine and inosine are both potent co-germinants for L-cysteine mediated germination whereas adenosine, xanthine, and xanthosine inhibit germination (Fig. 3a, b). The secondary interaction plot (Fig. 3c) further reveals non-parallel lines for the plots between hypoxanthine and inosine. Here, either hypoxanthine or inosine alone are sufficient as a co-germinant to trigger strong germination, and the extent of germination is not improved by both being present. Also notable are the less dramatic non-parallel graphs for hypoxanthine vs adenosine and hypoxanthine vs xanthosine. These show that the pro-germinant effect of hypoxanthine is slightly antagonized by adenosine and xanthosine.

To understand if these co-germinant effects were context-dependent, we tested these same 5 purines alone i.e., not in combinations (Supplementary Fig. S2c). Here, we found that hypoxanthine and inosine still showed strong co-germination effects, corroborating the earlier full factorial experiment. Additionally, however, adenosine also exhibited a significant co-germinant effect, in direct contrast to its inhibitory effect in the full factorial experiment. Such contextual effects for adenosine are not unprecedented. For example, adenosine inhibits inosine-triggered germination of *Bacillus cereus* spores while simultaneously acting as a co-germinant with L-alanine^30^. In the case of *C. novyi*-NT, it appears that adenosine may act as a strong co-germinant in the absence of other purines but perhaps not otherwise.

By comparing structures of the tested purines with germination activity, we observe that hypoxanthine, inosine, and adenosine share in common the reduced form of the purine ring, featuring a double bond between N3 and C2, which may be important for co-germinant activity (Fig. 3d). Xanthosine and xanthine on the other hand share the oxidized purine form, featuring a carbonyl at C2 which may account for their inhibitory activity. These observations are correlational and require further structure-activity studies to establish causality.

### D-alanine is the master inhibitor of germination

Germination receptors are often stereospecific in their recognition of L-amino acid germinants, with one stereoisomer triggering germination and the other inhibiting it. *Bacillus subtilis* is a classic example, where L-alanine induced germination is inhibited by D-alanine^31^. We wondered if germination by L-cysteine, L-alanine and L-valine could be antagonized by their stereoisomeric partners, possibly giving clues as to the hierarchy of germination signaling.

**Fig. 4:**
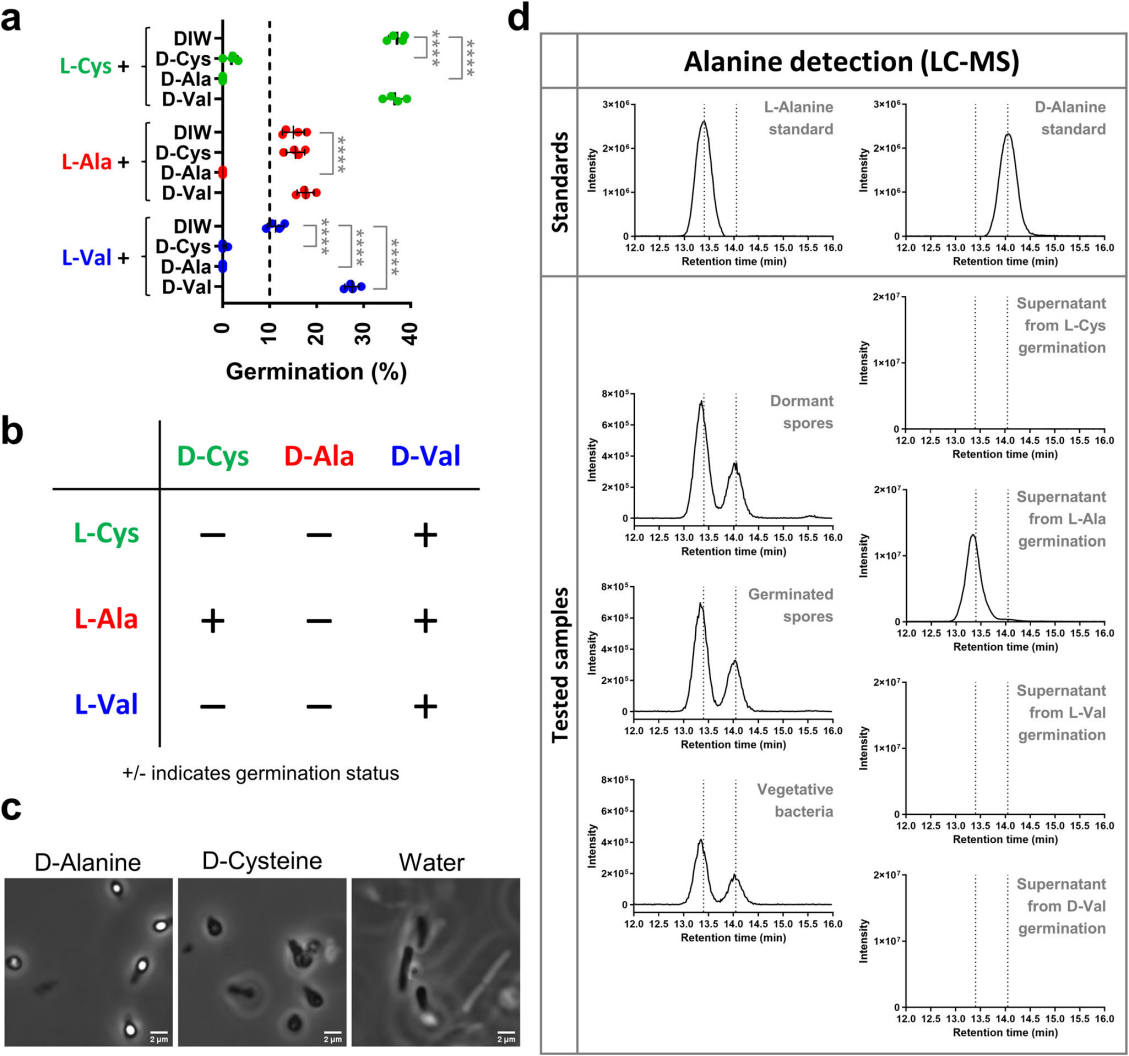
Inhibition of *C. novyi*-NT spore germination by D-alanine. **a** Scatter dot plot of percent germination (as defined in Supplementary Table 4) by L-amino acid germinants (91.5 mM) in the presence of candidate D-amino acid inhibitors (91.5 mM). Green, red and blue circles refer to L-cysteine, L-alanine, and L-valine, respectively. All data depict mean ± SD, *n* = 4 biologically independent samples. Significance at ****p ≤ 0.0001 (Unpaired t-test). **b** Summary table of results from (**a**). **c** Phase contrast images of spores inoculated overnight in RCM-FBS oxyrase media containing D-alanine (93.5 mM), D-cysteine (93.5 mM) and Water. Images are best representative from two independent experiments. Magnification 1600×, Scale bar 2 µm. **d** LC-MS EIC spectra of derivatized alanine ion (351 m/z) for Standards of L-alanine, D-alanine and Samples of peptidoglycan extracts from dormant spores, germinated spores, vegetative bacteria, and supernatants from spores germinated in L-cysteine, L-alanine, L-valine or D-valine. Dotted lines represent standard peaks for L-alanine and D-alanine at retention times of 13.4 and 14.05 min, respectively. Data shown is the best representative of water blank subtracted spectra from two independent experiments.

To answer this question, germination responses to these three factors were measured in the presence or absence of D-cysteine, D-alanine, and D-valine. All germinants were strongly inhibited by D-alanine, showing that these germinants acted through signaling pathways and not physicochemical stimulus (Fig. 4a, b). D-cysteine also showed an inhibitory effect, but for a subset of germinants (L-cysteine and L-valine). D-alanine’s inhibitory effect was effective even in complex media, demonstrating its potential role as a master inhibitory switch. (Fig. 4c). D-cysteine by comparison, allowed germination in complex media. In cases where D-alanine inhibits spore germination in other bacteria, it is often present in spore peptidoglycan or released during germination as a way to minimize premature germination, a phenomenon referred to as autoinhibition^32,33^. LC-MS analysis showed this to be the case with *C. novyi*-NT, with D-alanine present in the peptidoglycan of dormant and germinated spores, as well as vegetative bacteria though it wasn’t actively released into the media during germination. (Fig. 4d). Images of spores used in the LC-MS analysis are shown in Supplementary Fig. S3.

The final candidate inhibitor, D-valine, inhibited none of the germinants, and quite the opposite, even appeared to add to the baseline germination induced by L-valine (Fig. 4a).

### D-valine and its analogs are potent germinants

**Fig. 5:**
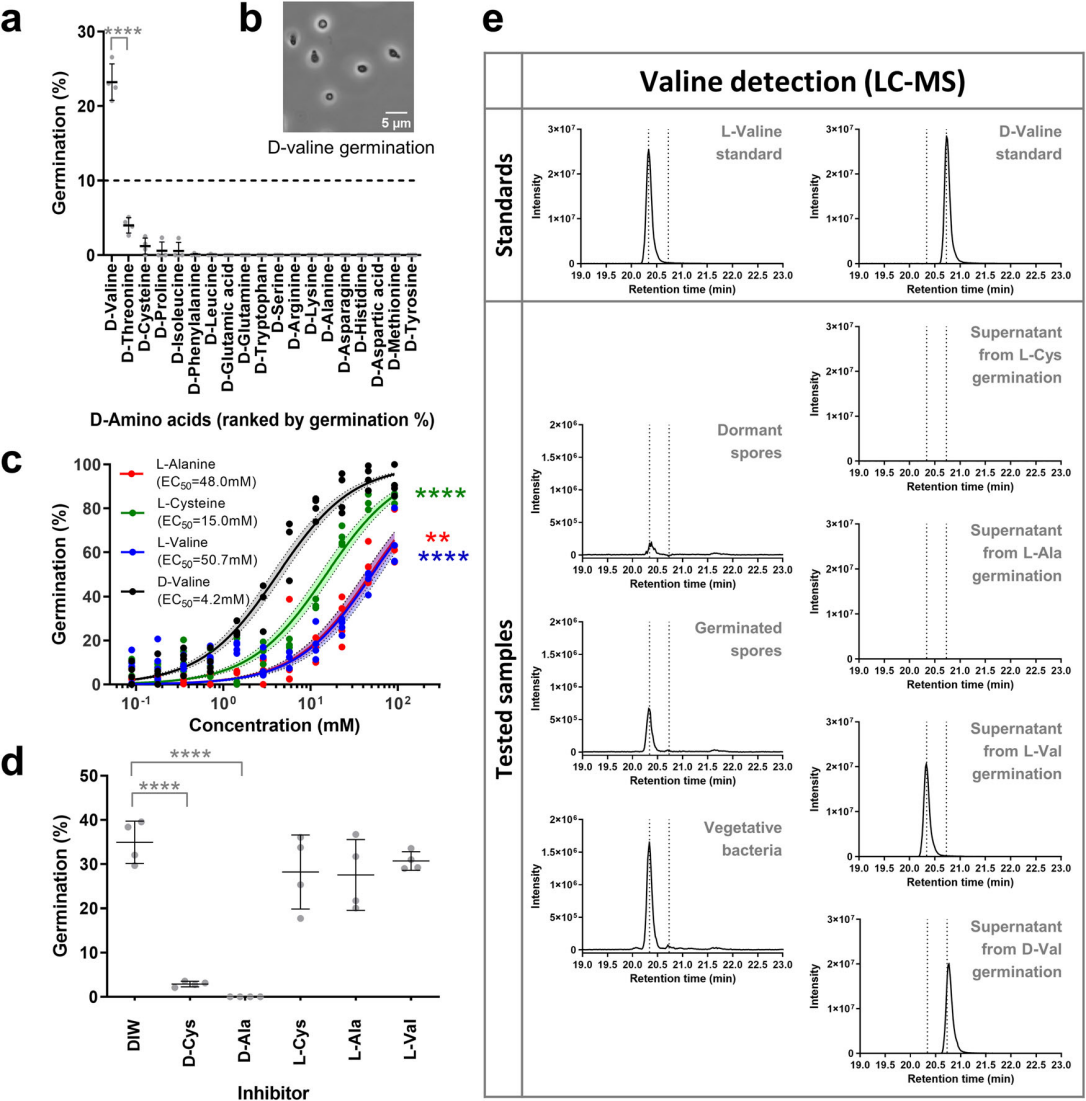
D-valine is a bona fide germinant. **a** Single factor screen for 19 D-amino acids where each D-amino acid was tested individually for germination at concentrations in Supplementary Table 3. Dashed line represents a germination threshold of 10%. D-amino acids are sorted in decreasing order of germination % from left to right. Significant at ****p ≤ 0.0001 (unpaired t-test) when compared with D-threonine. **b** Phase contrast image of *C. novyi*-NT spores germinated with D-valine (45.7 mM) at 1000× magnification. Scale bar 5 µm. **c** Dose response curve for germination response at different concentrations of D-valine (black), L-cysteine (green), L-alanine (red), and L-valine (blue) normalized to germination with D-valine at the highest concentration 91.5 mM. Circles depict individual values. Solid lines depict the least squares fit regression curve. Shaded area shows the 95% confidence error bands for each L-amino acid fit. EC_50_ values are shown in the legend and are significant at ****p ≤ 0.0001,***p ≤ 0.001,**p ≤ 0.01 (unpaired t-test) when compared to D-valine. **d** Germination by D-valine (91.5 mM) in the presence of other D- and L-amino acids (91.5 mM). Significant at ****p ≤ 0.0001 (unpaired t-test) when compared with DIW control. **e** LC-MS EIC spectra of derivatized valine ion (379 m/z) for Standards of L-valine, D-valine, and Samples of Peptidoglycan extracts of dormant spores, germinated spores, vegetative bacteria and supernatants from spores germinated in L-cysteine, L-alanine, L-valine or D-valine. Data shown is the best representative of water blank-subtracted spectra from two independent experiments. Dotted lines represent the standard peaks for L-valine (20.34 min) and D-valine (20.73 min) respectively. All germination data depict mean ± SD, *n* = 4 biologically independent samples. Germination % for all graphs are defined in Supplementary Table 4.

Intrigued by the D-valine results, we performed a single factor germination screen on a panel of 19 D-amino acids. To our surprise, D-valine could indeed trigger *C. novyi*-NT spore germination and was the only D-amino acid which was able to do so (Fig. 5a). D-valine germinated spores appeared phase dark under phase contrast microscopy, hence confirming this initial result (Fig. 5b).

The kinetics of D-valine germination, as measured by OD and Dipicolinic Acid (DPA) release, was similar to L-cysteine and L-alanine (Supplementary Fig. S4). It also turns out that significantly lower concentrations of D-valine were required to trigger germination compared with either L-cysteine or L-alanine, showing that D-valine was more potent as a germinant (Fig. 5c). Just like L-cysteine and L-valine, D-valine germination could be inhibited by both D-cysteine and D-alanine, providing further evidence that D-valine is a bona fide germinant (Fig. 5d).

The observation that D-valine can independently trigger *C. novyi*-NT germination goes against the general intuition that L-amino acids promote germination while D-amino acids inhibit it. D-valine specifically has not previously been shown to play any active role in bacterial spore germination. Could D-valine play a role as a signaling molecule and if so, what was the source of this D-valine? Since most bacteria incorporate D-amino acids (mostly D-alanine, D-glutamine, and sometimes D-valine) into their peptidoglycan during the stationary phase^34^, we wondered if D-valine could be a component of the spore cortex peptidoglycan layer^34–37^. To address this question, the peptidoglycan layer of dormant spores, germinated spores, and vegetative bacteria was extracted, hydrolyzed, and analyzed using derivatization LC-MS (Fig. 5e and Supplementary Fig. S3). This analysis revealed that D-valine was not present in the peptidoglycan of the spore or vegetative forms, even though L-valine was present in both (Fig. 5e). Further, D-valine was absent from the supernatant of L-cysteine or L-alanine or L-valine germinated spore samples (Fig. 5e and Supplementary Fig. S3), showing that D-valine was neither a constituent of the spore peptidoglycan, nor released during germination. It is conceivable that there could be a D-valine-specific racemase in the *C. novyi*-NT spores that generates L-valine. However, L-valine was not detected in spores germinated with D-valine, making this unlikely (Fig. 5e).

**Fig. 6:**
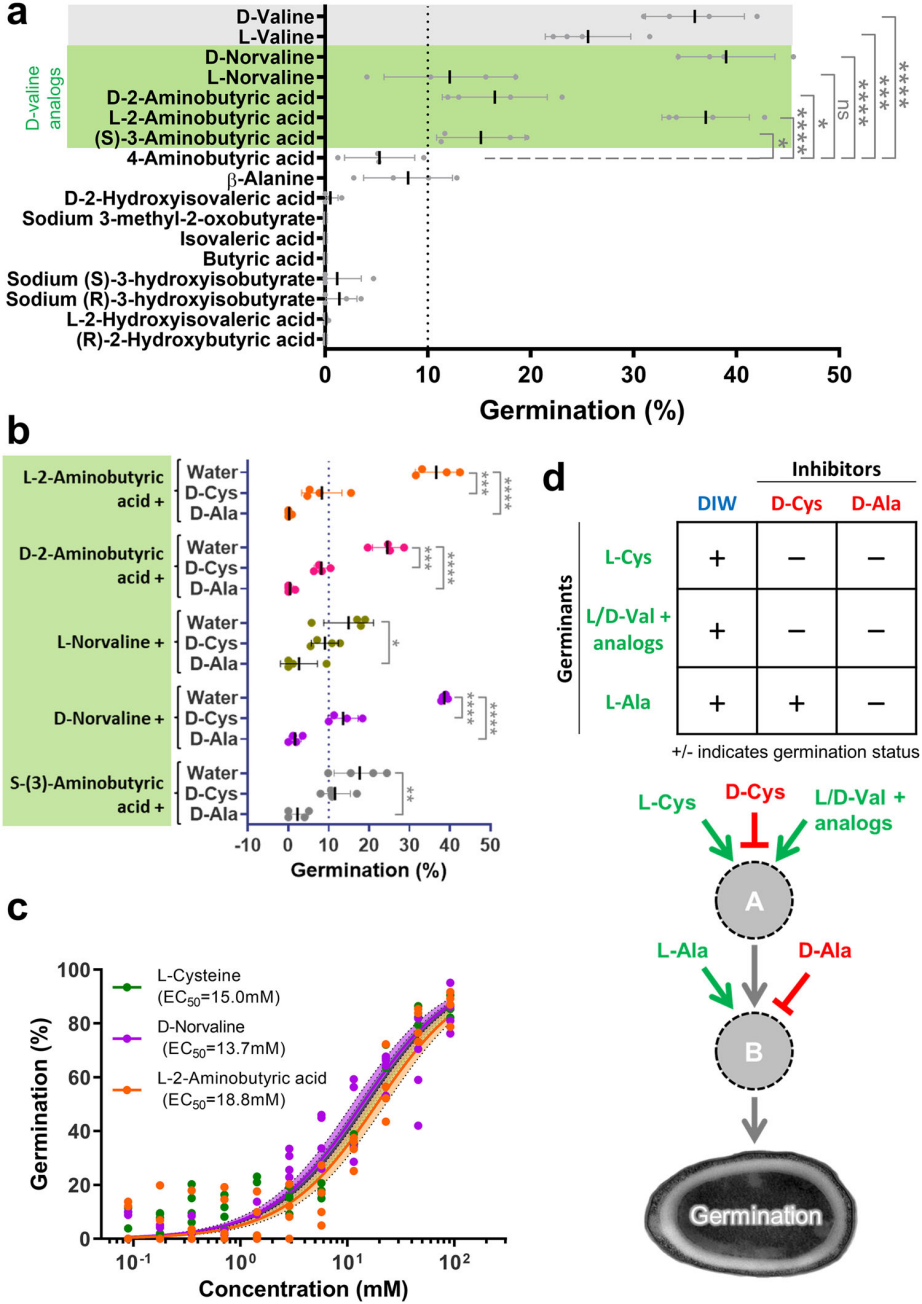
Germination with D-valine analogs. **a** Single factor screen of stereoisomeric structural analogs of D-valine. All analogs were at 91.5 mM concentration. D-valine and L-valine were used as reference positive controls (gray). All valine analogs with germination >10% (green) were identified as germinants. Significant at ****p ≤ 0.0001,***p ≤ 0.001,**p ≤ 0.01,*p ≤ 0.05, ns p > 0.05 (unpaired t-test) when compared to 4-aminobutyric acid. **b** Germination response of valine analogs (91.5 mM) L-2-aminobutyric acid (orange circles), D-2-aminobutyric acid (pink circles), L-norvaline (dark green circles), D-norvaline (purple circles), S-(3)-aminobutyric acid (gray circles) in the presence of the inhibitors D-cysteine and D-alanine (91.5 mM concentration). Significant at ****p ≤ 0.0001,***p ≤ 0.001,**p ≤ 0.01,*p ≤ 0.05, ns p > 0.05 (unpaired t-test) when compared to corresponding water controls. **c** Dose response curve for germination response at different concentrations of L-2-aminobutyric acid (orange), D-norvaline (purple) and L-cysteine (green) (normalized to germination with D-valine at the highest concentration 91.5 mM) shows potency of valine analogs similar to L-cysteine. Circles depict individual values. Solid lines depict the least squares fit regression curve. Shaded area shows the 95% confidence error bands for each amino acid fit. EC_50_ values are shown in the legend. **d** Proposed two-component signaling framework for *C. novyi*-NT germination. All germination data depict mean ± SD, *n* = 4 biologically independent samples. Germination % for all graphs are defined in Supplementary Table 4.

Since the spore was shown not to be a source of D-valine, and given the presumed scarcity of D-valine in mammalian tissues colonized by *C. novyi*-NT, we asked if an analog of D-valine might be the actual relevant germinant. We screened a collection of D-valine analogs (Supplementary Fig. S5) and discovered 5 analogs that showed germinant activity (Fig. 6a). Interestingly, 4 of these analogs comprised two pairs of stereoisomers; L-norvaline and D-norvaline, as well as L-2-aminobutyric acid (L-AABA) and D-2-aminobutyric acid (D-AABA). These two pairs mirror our observation with valine, with both L and D enantiomers showing germinant activity. Since D-alanine and D-cysteine had been previously shown to inhibit germination by L- and D-valine, we performed the same experiment and observed that D-alanine and D-cysteine were also able to inhibit germination induced by the valine analogs (Fig. 6b). This suggests that these analogs act through the same signaling pathway as L- and D-valine, possibly acting through the same germinant receptor. L-AABA and D-norvaline, the strongest germinants among the analogs, have similar potency to L-cysteine (Fig. 6c).

We wondered if perhaps valine, AABA and norvaline were metabolites in vegetative *C. novyi*-NT. Metabolites from germinated rod forms could theoretically serve as germinants to further drive the germination of spores. LC-MS analysis for valine and norvaline were performed on rods grown in liquid medium and showed that both were absent from pelleted vegetative *C. novyi-*NT and media conditioned by its growth (Supplementary Fig. S6a). For technical reasons, the same LC-MS analysis for AABA was performed on colonies grown on agar medium. Here, it was observed that L-AABA was present in *C. novyi*-NT rods, but D-AABA was absent (Supplementary Fig. S6b). Besides being a putative metabolite of *C. novyi*-NT, L-AABA is also a non-specific marker of sepsis^38^, as well as a metabolite produced by other similar necrotizing clostridia such as *C. botulinum* and *C. sordelli*^39,40^, hence also implicating the environment as a potential source of L-AABA. These findings hint that D-amino acids and their analogs may play yet undiscovered roles in bacterial spore germination.

## Discussion

DOE has played an important role in this study. Only L-cysteine and L-alanine would have been identified as germinants in a single factor screen. Combining DOE with single factor screens led to the further discovery of pro-germinants and inhibitors which act only in combinations, and drove the sequence of investigations which identified D-valine and its analogs as bona fide pro-germinants. The effects of single factors acting independently can deviate from the effects of factors in combination, and hence we hope that this tool will find more common use in bacterial spore germination studies. Especially, such interactions between factors would exist in the real world inhabited by the spore and DOE represents an efficient way to probe and understand the combinatorial space more efficiently.

In this first use of DOE in a screen for bacterial spore germinants, we have identified the main germinant effects for *C. novyi*-NT. While a full pathway cannot yet be fully described, there is sufficient information to infer a rudimentary framework for the sequence of germination signaling events (Fig. 6d). This purely hypothetical framework is based on the summary table of factors in Fig. 6d, and will have to be revised as more experimental data is available. In this framework, there are two placeholder elements A and B, which aggregate signals from germinants and inhibitors. Here, B is downstream of A, and germination is downstream of B. Since D-alanine inhibits all germinants, it is more likely to act on B, which is the most downstream element. D-cysteine’s inhibition by contrast, isn’t absolute, and hence we propose that it might act upstream on element A. Using similar reasoning, the germinants inhibited by D-cysteine (L-cysteine; L/D-valine and their analogs) would also act upon the same element A. Finally, since L-alanine escapes the inhibition of D-cysteine but not D-alanine, it may act on the downstream element B.

A and B are placeholders for participants in germinant signaling which have not yet been identified. Both could represent either single or multiple signaling participants, and these participants could possibly derive from two sets of germination operons (NT01CX_0189-0191 and NT01CX_0562-564) within the *C. novyi*-NT genome^41^. In particular, NT01CX_0191, NT01CX_0562, and NT01CX_0564 show high sequence similarities to germinant receptor family genes in *B. cereus* and *C. botulinum* (Supplementary Table 6), and are, therefore interesting candidates for future investigation.

Among all the germinant classes, only the L-amino acids are universally featured in all known bacterial spores as pro-germinant factors. The recognition of amino acid germinants is facilitated by Ger-type receptors, which are present in almost all sporulating *Clostridium* and *Bacillus*^42^. One rare exception to this rule is *C. difficile,* which uses the Csp type receptor (CspA)^43,44^. Unsurprisingly, all germinant studies involving *Clostridium* have thus identified at least one amino acid as a pro-germinant (i.e., either a germinant or co-germinant)^45^. How do the amino acid pro-germinant factors for *C. novyi*-NT compare with other clostridia? In a survey of 14 clostridia (including *C. novyi*-NT from this study), L-alanine (10/14) was the most common pro-germinant followed by L-cysteine (7/14) (Supplementary Table 7). *C. novyi*-NT follows this popular trend by using these two L-amino acids as pro-germinants. In addition to these two L-amino acids, clostridia also feature other L-amino acids as pro-germinant factors at a lower frequency. *C. novyi*-NT is no different and exhibits a germination response to L-valine. *C. novyi*-NT also germinates in response to non-canonical germinants which are not classical L-amino acids, such as L-norvaline and L-2-aminobutyric acid. In this sense, they are similar to *C. frigidicarnis* which responds to L-norvaline^46^, and *C. difficile* which responds to both L-norvaline and L-2-aminobutyric acid^47^. L-2-aminobutyric acid may have metabolic significance as a nutritive germinant since it is produced as an intracellular metabolite in vegetative *C. novyi*-NT. One possibility is that L-2-aminobutyric acid germination acts as positive feedback from early germinating spores to encourage further germination. Further, the structural similarity of L-2-aminobutyric acid to L-norvaline and L-valine means that all three might bind to the same germination receptor (Supplementary Fig. S5). Further research is needed to shed light on these hypotheses.

To date, practically all amino acid pro-germinants reported for clostridia are L-amino acids. The only clostridia to deviate from this trend are *C. bifermentans* (which uses D-alanine, D-arginine, and D-serine as co-germinants)^48,49^, *C. difficile* (which uses D-alanine or D-serine as a co-germinants)^50^, and with this study, *C. novyi*-NT (which germinates in response to D-valine and its analogs). Since *C. bifermentans*, *C. difficile,* and *C. novyi*-NT are the only clostridia to our knowledge where the D-amino acid panels have been screened for pro-germinant activity, the true incidence of D-amino acid germinants among the clostridia is very likely to be underestimated.

It is a common occurrence that D-amino acids may act as germination inhibitors, because individual L-amino acid germinants are usually antagonized by their D-amino acids mirror images. We were hence surprised to find not only that D-valine failed to antagonize L-valine germination, but that D-valine turned out to be an even more potent germinant than L-valine. Another unexpected observation was that D-alanine prevented spores from germinating in Reinforced Clostridial Medium, a complex rich culture medium. Empirically, D-amino acids may inhibit a wider range of germinants than just their L-amino acid counterparts. Taking *C. sporogenes* as an example, D-serine, has been shown to inhibit germination to L-serine, and additionally, L-cysteine, L-methionine and L-phenylalanine^51^. D-alanine, which has similarly been shown to inhibit germination in *Bacillus thiaminolyticus* to L-glutamine, L-norvaline, L-serine, L-methionine and L-threonine, is no different^52^. Despite these examples, no previous study has shown the seemingly absolute inhibition of germination as observed with D-alanine in this study. One possibility could be the dearth of studies testing D-alanine inhibition in rich complex media. Another explanation is that germination signaling in *C. novyi*-NT may converge on a single gatekeeper whereas the nature of germinant signaling may be more parallel in other clostridia, with multiple redundant paths to triggering germination.

While the specifics of *C. novyi*-NT germination remain to be fleshed out, two broad themes are highlighted by this work. Firstly, germination factors can either act with a high degree of independence (like L-cysteine and D-alanine), or interdependently (like L-alanine or L-threonine). While we have grown accustomed to thinking of spore germinants as lone actors, our data demonstrates that the germinant properties of certain amino acids may emerge or disappear depending on the presence of other amino acids. The PB screen in our study can show the existence of interactions, but it is not designed to explore these interactions in great detail. More work hence needs to be done to elucidate the nature of these interactions.

Stereoflexibility is the second theme. Nature is picky about chirality, and thus, spore germinant receptors ordinarily recognize specific L-amino acids as their cognate germinants. The D-amino acid enantiomers to these germinants may on the other hand interrupt these interactions, hence inhibiting germination^51^. This simple and elegant homochiral model may have to be revisited in light of new research showing that D-serine and D-alanine may act as co-germinants for *C. difficile*^50,53^. In that research, genetic inactivation of *alr2*, an alanine racemase, abrogated co-germinant activity of these D-amino acids, showing that conversion to the L form was essential for germinant activity. Although a racemase activity involving the conversion of D-valine to L-valine racemase activity could theoretically explain *C. novyi*-NT’s stereoflexibility, such racemase activity was not detected in this study. Alternatively, it is also possible that there is a germinant receptor in *C. novyi*-NT which binds to amino acids in a stereoflexible manner. There is certainly a need for more investigation to disambiguate between these possible explanations. Unlike *C. difficile*, D-valine and its analogs act not as co-germinants but as primary germinants in *C. novyi*-NT, with germination effects rivaling L-cysteine, the main L-amino acid germinant. This data poses even stronger evidence that the homochiral model is incomplete.

Reasoning from first principles, the germinant vocabulary of a spore-forming bacterium determines the niche which it inhabits. Hence, it is reasonable to expect that germinant sensing is not based on a few key nutrients but on a multiplexed sensing of the environment. Complex interactions and stereoflexible sensing may be the keys which open the door to the true germinant vocabulary of bacterial spores. They may also lay the early foundation for understanding and improving *C. novyi*-NT’s tumor-colonizing ability.

Solid tumors are poorly vascularized and contain regions of hypoxia and necrosis^54^. This property correlates with poorer outcomes for radiotherapy and chemotherapy, but also presents a therapeutic opportunity for spores of strictly anaerobic clostridia to colonize and kill cancer cells^55^. Not all clostridia are however created equal for this purpose. Although all are obligate anaerobes, only two out of eight strains tested (*C. novyi*-NT and *C. sordelli*) were found in one study to effectively colonize the hypoxic regions of experimental tumors^4^.

One factor which could influence colonization efficiency is the extent to which key germinants are present in the tumor microenvironment. Several pro-germination factors of *C. novyi*-NT are in this respect well-enriched in solid tumors. Intratumoral L-cysteine for example has been shown to reach up to 1.40 mM in certain tumors^56^, exceeding the average baseline of ~0.2 mM in plasma^57^. Increased intratumoral cysteine levels are thought to protect cancer cells from the effects of radiotherapy^58^ and chemotherapy^59^, but they may also potentially promote *C. novyi*-NT germination within these treatment-resistant niches. In fact, *C. novyi*-NT requires just 1 mM of L-cysteine in combination with either co-germinant (hypoxanthine or inosine) to achieve weak germination (Supplementary Fig. S2d).

Besides L-cysteine, the co-germinants, hypoxanthine and inosine, are similarly enriched in tumors^60,61^. Both are substrates in the purine metabolic pathways which drive cancer cell proliferation, and are released from dying cells in necrotic and putrefying tissue. Hypoxanthine is hitherto not known to promote the germination of bacterial spores. Inosine is, on the other hand, associated with other tissue-colonizing bacteria either as a germinant (*B. cereus*^29^, *Bacillus anthracis*^62^), or co-germinant (*Clostridium botulinum* type E^63^). Taken together, tumor concentrations of L-cysteine, hypoxanthine and inosine are likely to have an important effect on colonization and could contribute to the variation in germination observed between different cancer patients treated with *C. novyi*-NT spores^64^.

Now that we have a fuller picture of how *C. novyi*-NT spores germinate, we can potentially induce the germination of spores in previously non-responsive tumors, hence achieving more consistent results across different solid tumors. Also, engineering strains of *C. novyi*-NT which are more responsive to one or more of its germinants may further increase the therapeutic effect of this promising cancer therapeutic.

## Methods

### Materials

All L-amino acids, D-amino acids (except D-histidine), purines, Valine analogs, Terbium chloride (212903), Maltose (M5895), Sodium phosphate dibasic (S7907), o-phthaldialdehyde (P0657), N-acetyl cysteine (A7250), Sodium tetraborate decahydrate (B3545), HPLC grade methanol (34860), 1-Propanol (34871), Trifluoroacetic acid (302031) and Mutanolysin enzyme (M9901) were purchased from Sigma. 2-Propanol (29113-95) was obtained from Nacalai Tesque. D-Histidine (sc-255057) was purchased from SantaCruz Biotechnology. Acetonitrile (1.00317) and HCl (1.00029) were purchased from Merck. Percoll (17089109) was purchased from GE Healthcare. Oxyrase for broth was purchased from Oxyrase Inc and Sigma (SAE0013). BBL polypeptone peptone (211910), Dehydrated cooked meat media (226730), Brain heart infusion broth (BHI, 237500) and Reinforced Clostridial medium (RCM, 218081) were purchased from BD. Fetal bovine serum (S1810) was obtained from iDNA Biotechnology.

*C. novyi*-NT was a gift from the Kinzler-Vogelstein lab in John Hopkins University, USA.

### *C. novyi*-NT sporulation

*Clostridium novyi*-NT spores were prepared using the protocol from *Cheong* et al.^5^. Briefly, 200 µl of 5 × 10^9^ CFU/ml of *C. novyi*-NT spores were inoculated in 1 l of polypeptone and cooked meat rich sporulation media (L-cysteine 0.5 g/l, Sodium phosphate dibasic or Na_2_HPO_4_ 5 g/l, BBL polypeptone peptone 30 g/l, Dehydrated cooked meat media 50 g/l, Maltose 10 g/l and FBS 10% v/v) and the culture was sporulated inside a sealed BD GasPak^TM^ anaerobic jar and incubated at 37 °C for three weeks. Spores were harvested by resuspending the pellet in 1X PBS and separating the spores from vegetative cells using 90% Percoll solution at 15000 rcf for 30 min in a Beckman Avanti J-20 XP high performance centrifuge. Spore quality was checked by phase contrast microscopy and was found to contain >99% phase bright spores. The spore concentration was adjusted to 5 × 10^9^ CFU/ml by appropriate dilution in 1× PBS using a standard curve correlating OD600 absorbance to cell count.

### Spore germination assays

All spore germination experiments were performed anaerobically using oxyrase for broth enzyme in a Greiner Bio-One 384 clear microwell plate (#781186). Anaerobic conditions by itself did not trigger any germination. To 50 µl of germinant solution containing oxyrase (1:50 v/v) and germinants in different combinations as described below, 3.5 µl of 5 × 10^9^ CFU/ml *C. novyi*-NT spores in 1× PBS were added. The plate was made airtight with a Sealplate sealing film and incubated at 37 °C overnight. The OD600 reading was measured using a Tecan Spark® multimode microplate reader every 10 min through the Spark Control Dashboard V2.2 software.

All end-point germination responses were calculated at approximately time *t* = 7.3 h. All the DOE screening design analyses were performed using Minitab 20. The model fit parameters for all the DOE experiments are shown in Supplementary Table 8. The germination response for all the DOE experiments and single factor-based germination experiments were calculated as specified in Supplementary Table 4.

### Plackett-Burman screen for L-amino acids

The Plackett-Burman design is a two-level fractional factorial screening design that uses high and low level concentrations for studying N-1 variables using N combinations, where N is a multiple of 4. For the 20 L-amino acids, a 24 combination Plackett-Burman design using Minitab19 was generated as shown in Supplementary Table 1 and was set up in a 384-well microtiter plate using the automated workstation Biomek i5, where each well consisted of a particular combination of all the 20 L-amino acids prepared from a mastermix according to each row in Supplementary Table 1. All 20 L-Amino acids were prepared at high stock concentrations, which when diluted by a factor of 20 and mixed with the spores gave final concentrations (high level) depicted in Supplementary Table 3. The low level concentration was set to zero.

### Single factor screens

For the L-amino acid single factor screen, each well contained only one amino acid, and the final concentrations were twice that of the PB assay (Supplementary Table 3). The D-amino acid single factor screen was also performed similarly, but using the final concentrations when mixed with spores as shown in Supplementary Table 3. For the D-valine analogs, the final concentration when mixed with spores was 91.5 mM. The final concentration of purines when mixed with spores in the co-germinant single factor screens was 0.1 mM, while the final L-cysteine levels added to each well when mixed with spores was 10 mM. For the physiological level germination experiment, purines and L-cysteine were used at final concentrations of 0.1 and 1 mM respectively.

### Co-germinant screen for purines and L-Amino acid interaction study

A full factorial design as depicted in Supplementary Table 2 was used for studying germination response with purines and for the L-amino acid interaction study. For combinatorial germinant screen and combinatorial co-germinant screen with purines, stock solutions of 0.6 mM of respective purines in 0.005 N NaOH were prepared and diluted (final concentration 0.1 mM) in the absence and presence of L-cysteine (final concentration 10 mM) respectively with each well representing one combination of the factorial design. For the L-amino acid interaction studies, L-cysteine and the positive hits from the PB and single factor screen and the negative hits from the PB screen were used respectively. The concentrations used were the same as in Supplementary Table 3. All assays were set up on a 384-well microtiter plate using the Biomek i5 automated workstation.

### Inhibition studies with D-amino acids and dose response experiments

For the germination inhibition studies, equal volumes of the germinant with the enantiomeric inhibitor were added so that the final concentrations of both when mixed with spores were 91.5 mM each. For the dose response experiments, stock solutions of germinants at 100 mM were serially diluted two-fold in deionized water up to a concentration of 90 µM. The final concentrations when mixed with spores ranged from 91.5 mM to 89 µM.

### DPA assay

For the dipicolinate release assay, all germinants were prepared at concentration of 200 mM and diluted to a final concentration of 100 mM in a mixture containing 0.1 mM Terbium chloride and oxyrase (1:50 v/v). To 50 µl of this solution, 3.5 µl of *C. novyi*-NT spores were added in a Greiner Bio-One 384 UV star microtiter plate. The final concentrations after addition of spores were 93.5 mM for germinants and 0.09 mM for terbium. The OD600 reading and the Fluorescence at 545 nm (lag time of 20 µs) were measured using a Tecan Spark® multimode microplate reader every 10 min through the Spark Control Dashboard V2.2 software.

### D-amino acid outgrowth inhibition assay

To RCM-FBS media containing oxyrase (1:50 v/v), corresponding D-amino acid or water were added followed by addition of *C. novyi*-NT spores in 1.5 ml eppendorf tubes. Final concentrations of components were 93.5 mM D-amino acids, 0.35× RCM, 9.3% FBS. The culture tubes were then incubated overnight at 37 °C and aliquots of the overnight culture were then imaged.

### Phase contrast microscopy

Aliquots (10 µl) of overnight germination/outgrowth experiments were added on to a Superfrost plus microscope slide, covered with #1.5 microscope cover slips and then sealed with nail polish. The slides were then imaged on a Zeiss inverted Axio Observer 7 microscope using a 100×/1.4 Ph3 Plan APOCHROMAT Objective set at an Optovar setting of 1× or 1.6× magnification as specified. The images were captured using a Hamamatsu CMOS camera and Metamorph software (Molecular devices, 7.10.3) with an exposure time of 100 ms in the Phase contrast mode. The images were cropped using ImageJ Version 1.53n.

### Peptidoglycan extract sample preparation

The protocols for peptidoglycan extraction were adapted from Atrih et al. (1996,1998) and Popham et al. (1996) for spores and Atrih et al. (1999) for vegetative bacteria.

Briefly, 3 ml of *C. novyi*-NT spores (5 × 10^9^ CFU/ml) were germinated at 37 °C for 30 min in a germinant solution containing L-cysteine (100 mM), hypoxanthine (0.1 mM) and Oxyrase (1:50 v/v) which when diluted with spores gave a final concentration of L-cysteine (82 mM) and hypoxanthine (0.08 mM). Equivalent amounts of dormant spores in 1X PBS and germinated spores in deionized water were re-suspended in 1 ml of Propan-2-ol and Propan-1-ol respectively and subjected to heat treatment at 85 °C for 15 min. This was followed by centrifugation at 14000 × *g* for 8 min using a Beckman Coulter Microfuge 22R centrifuge. The pellets were resuspended in 13 ml and 1 ml of 50 mM Tris pH 7.4–4% SDS–30 mM DTT-2mM EDTA buffer for dormant and germinated spore samples respectively. The samples were then heated at 100 °C for 16 min and then at 37 °C for 40 min.

For vegetative bacteria extract, 3 tubes of overnight *C. novyi*-NT culture (5 ml per tube) were combined and resuspended in 1 ml of deionized water. The sample was then heated at 85 °C for 15 min and centrifuged at 14,000 × *g* for 8 min. The pellet was resuspended in 10 ml of 5% SDS and heated at 100 °C for 25 min. The sample was centrifuged at 14,000 × *g* for 8 min and resuspended in 1 ml of 5% SDS and again heated at 100 °C for 15 min.

Following the respective heat treatments, the spore and vegetative bacteria samples were centrifuged at 14000 × *g* for 8 min and the pellets were washed with 37 °C pre-warmed de-ionized water five times. The supernatant after the final spin was discarded and the pellets were treated with 250 µl of Mutanolysin enzyme (125 U/ml in 12.5 mM sodium phosphate buffer pH 5.8) for 16 h at 37 °C. The samples were then centrifuged at 14,000 × *g* for 8 min and the supernatant was freeze-dried overnight. The freeze-dried pellets were then subjected to hydrolysis in 1:1 6N HCl: Trifluoroacetic acid (v/v) in a Thermofisher vacuum hydrolysis tube (Cat no. 29570) and heated at 160 °C for 1 h in an oil bath. The acid was evaporated using an Eppendorf concentrator overnight and the pellets were resuspended in 200 µl in deionized water and sent for further analysis.

### Metabolite extract sample preparation

For aminobutyric acid analysis, *C. novyi*-NT was grown overnight in a Plas labs anaerobic chamber in 5 ml of BHI-10%FBS media. 100 microlitre of the overnight culture was plated on a BHI-FBS agar plate while the control plates were streaked with BHI-10%FBS media. The colonies were harvested the next day using 1× PBS (500 µl). The solutions were centrifuged at 5000 rcf, 5 min, 4 °C using a Beckman Coulter Microfuge 22R centrifuge. The pellet was resuspended in 50% Acetonitrile and homogenized using the Biospec Mini bead beater 24 (3500 rotations/min, 1 min, 4 times). The samples were then centrifuged at 16,000 rcf for 10 min at 4 °C. The supernatants were freeze-dried and resuspended in 10% Acetonitrile and sent for further analysis.

For Norvaline and Valine analyses, *C. novyi*-NT was grown overnight in a Plas labs anaerobic chamber in 5 ml of BHI-10%FBS media. The cultures were centrifuged at 4700 rcf, 5 min, 4 °C using a Thermo Scientific Sorvall ST 40R centrifuge, and the supernatant was collected for further analysis. The pellet was resuspended in 50% Acetonitrile and homogenized using the Biospec Mini bead beater 24 (3500 rotations/min, 1 min, 4 times). The samples were then centrifuged at 16,000 rcf for 10 min at 4 °C. The supernatant, pellet and media alone control samples were freeze-dried and resuspended in 10% Acetonitrile (pellet) or water (supernatant and media control) and sent for further analysis.

### Supernatant sample preparation

*C. novyi*-NT spores were germinated in 91.5 mM (final concentration with spores) of L-cysteine, L-alanine, L-valine, D-valine and water containing oxyrase enzyme (1:50 v/v) for 30 min at 37 °C. After 30 min, the germinated spores were pelleted down at 3900 rcf, 5 min and the supernatant from each sample was sent for further analysis.

### LC-MS analysis

All peptidoglycan extracts, metabolite extracts, and supernatant samples were analyzed using an Agilent 6546 LC/Q-TOF machine on a Phenomenex, Synergi 4 µm Fusion-RP 80 A° column using an Acetonitrile and 10 mM Ammonium formate/0.1% formic acid mobile phase gradient. Samples were derivatized using o-pthaldialdehyde and N-acetyl cysteine in methanol (10%)-sodium tetraborate buffer. The LC-MS EIC spectra were analyzed using MassHunter Software (Agilent, B.08.00) and Microsoft Excel.

### Statistics and reproducibility

DOE experiments were replicated twice with two embedded technical replicates. Single factor spore germination assays were performed with 4 biological replicates, except for Fig. 1c, where one biological replicate for L-asparagine was excluded for being a clear outlier. The criteria for outlier detection was pre-established as: [*x*_*i*_ − median(*x*_*i*_)]/(median of all absolute deviations from the median) at a cutoff of 2.5. Including the outlier does not change the result of the experiment. More than 3.2 × 10^8^ CFU/ml of spores were used for each germination experiment, while spores of the order of 10^9^ CFU/ml were used for the LC-MS experiments. This is far in excess of an amount which could cause error due to under-sampling. The statistical analyses for the combinatorial experiments (ANOVA) were performed using the Minitab 20 software while the same for the single factor experiments (Student’s t-test unpaired) were conducted using GraphPad Prism v8.4.3 with the exact p values provided in Supplementary Table 5. The means with standard deviations were also calculated using GraphPad Prism v8.4.3. Representative LC-MS spectra were determined based on at least two independent samples for each condition. Representative images were obtained based on at least two independent samples.

### Reporting summary

Further information on research design is available in the Nature Portfolio Reporting Summary linked to this article.

## Supplementary information

Supplementary Information

Description of Additional Supplementary Data

Supplementary Data

Reporting Summary

## Data Availability

All data generated or analyzed during this study are included in this published article (and its supplementary information files). Source data for figures can be found in Supplementary Data.
