# Supplementary material for: A design of experiments screen reveals that Clostridium novyi-NT spore germinant sensing is stereoflexible for valine and its analogs
Source: Commun Biol. 2023 Jan 28;6:118. doi: 10.1038/s42003-023-04496-9 (PMC9884283; doi:10.1038/s42003-023-04496-9)
Supplement: Supplementary file 1 — Supplementary Information [file 42003_2023_4496_MOESM1_ESM.pdf]

A Design of Experiments screen reveals that *Clostridium novyi*-NT spore germinant sensing is stereoflexible for valine and its analogs

Ajitha Sundaresan[1,2], Mai Le Ngoc[3,†], Marvell Ung Wew[3,†], Varsha Ramkumar[3,†], Prahlad Raninga[1], Rongji Sum[1,2], Ian Cheong[1,2]*

[1] Temasek Life Sciences Laboratory, Singapore, Singapore
[2] Department of Biological Sciences, National University of Singapore, Singapore
[3] NUS High School of Mathematics and Sciences, Singapore, Singapore.

[†] These authors contributed equally
*Correspondence to: ian@tll.org.sg

Plackett-Burman design used in initial screen

Amino Acid (single letter)

Combinations of amino acids	E	T	F	P	Q	G	S	R	K	A	L	I	V	C	N	H	D	M	Y	W
R1	+	+	+	+	+	+	+	+	+	+	+	+	+	+	+	+	+	+	+	+
R2	-	+	+	+	+	-	+	-	+	+	-	-	+	+	-	-	+	-	+	-
R3	-	-	+	+	+	+	-	+	-	+	+	-	-	+	+	-	-	+	-	+
R4	-	-	-	+	+	+	+	-	+	-	+	+	-	-	+	+	-	-	+	-
R5	-	-	-	-	+	+	+	+	-	+	-	+	+	-	-	+	+	-	-	+
R6	-	-	-	-	-	+	+	+	+	-	+	-	+	+	-	-	+	+	-	-
R7	+	-	-	-	-	-	+	+	+	+	-	+	-	+	+	-	-	+	+	-
R8	-	+	-	-	-	-	-	+	+	+	+	-	+	-	+	+	-	-	+	+
R9	+	-	+	-	-	-	-	-	+	+	+	+	-	+	-	+	+	-	-	+
R10	-	+	-	+	-	-	-	-	-	+	+	+	+	-	+	-	+	+	-	-
R11	-	-	+	-	+	-	-	-	-	-	+	+	+	+	-	+	-	+	+	-
R12	+	-	-	+	-	+	-	-	-	-	-	+	+	+	+	-	+	-	+	+
R13	+	+	-	-	+	-	+	-	-	-	-	-	+	+	+	+	-	+	-	+
R14	-	+	+	-	-	+	-	+	-	-	-	-	-	+	+	+	+	-	+	-
R15	-	-	+	+	-	-	+	-	+	-	-	-	-	-	+	+	+	+	-	+
R16	+	-	-	+	+	-	-	+	-	+	-	-	-	-	-	+	+	+	+	-
R17	+	+	-	-	+	+	-	-	+	-	+	-	-	-	-	-	+	+	+	+
R18	-	+	+	-	-	+	+	-	-	+	-	+	-	-	-	-	-	+	+	+
R19	+	-	+	+	-	-	+	+	-	-	+	-	+	-	-	-	-	-	+	+
R20	-	+	-	+	+	-	-	+	+	-	-	+	-	+	-	-	-	-	-	+
R21	+	-	+	-	+	+	-	-	+	+	-	-	+	-	+	-	-	-	-	-
R22	+	+	-	+	-	+	+	-	-	+	+	-	-	+	-	+	-	-	-	-
R23	+	+	+	-	+	-	+	+	-	-	+	+	-	-	+	-	+	-	-	-
R24	+	+	+	+	-	+	-	+	+	-	-	+	+	-	-	+	-	+	-	-

Supplementary Table 1. **Plackett Burman design with 24 combinations for 20 L-amino acids**. Rows indicated as R1-R24 represent each combination while columns represent the presence (+) or absence (-) of a single amino acid in each combination. L-amino acids are depicted by their single letter nomenclature and used at concentrations indicated in **Supplementary Table 3** when present.

Full Factorial experimental designs used in this study

Relevant Figure	Class of factors tested		Factors tested				
Figs. 2a-c	Amino acid (single letter)		C	V	T	H	A
Figs. 2d-f	Amino acid (single letter)		C	G	K	R	W
Figs. 3a-c,Suppl. Figs. S2a,b	Purines (3-letter)		Hyp	Ino	Ado	Xao	Xan
Combinations of factors		**R1**	+	-	+	+	+
		**R2**	+	+	-	-	-
		**R3**	-	+	-	-	-
		**R4**	+	+	+	+	-
		**R5**	-	+	+	-	+
		**R6**	-	-	-	+	-
		**R7**	-	-	-	-	+
		**R8**	+	+	+	-	-
		**R9**	+	+	-	-	+
		**R10**	-	-	+	-	+
		**R11**	-	+	-	+	-
		**R12**	-	-	+	+	+
		**R13**	+	+	+	+	+
		**R14**	-	-	+	+	-
		**R15**	+	+	-	+	-
		**R16**	-	-	-	+	+
		**R17**	-	+	+	+	+
		**R18**	+	-	+	-	-
		**R19**	-	+	+	+	-
		**R20**	+	-	-	-	-
		**R21**	+	+	-	+	+
		**R22**	+	-	-	+	-
		**R23**	+	-	-	-	+
		**R24**	+	+	+	-	+
		**R25**	+	-	+	+	-
		**R26**	-	+	-	-	+
		**R27**	+	-	-	+	+
		**R28**	-	+	-	+	+
		**R29**	-	-	-	-	-
		**R30**	-	-	+	-	-
		**R31**	-	+	+	-	-
		**R32**	+	-	+	-	+

Supplementary Table 2. Full factorial design with 32 combinations for five factors. Rows of the matrix labeled R1-R32 represent each combination while columns represent the presence (+) or absence (-) of a single factor (amino acid or purine). Amino acids are indicated by their single letter nomenclature while for purines the three-letter code was used as follows - Hyp-hypoxanthine, Ino-Inosine, Ado-Adenosine, Xao-Xanthosine, Xan-Xanthine. Factors are used at concentrations as depicted in **Supplementary Table 3** when present.

CANONICAL AMINO ACIDS	L-amino acid		D-amino acid	
	**Cat. No.**	**Conc (mM)**	**Cat. No.**	**Conc (mM)**
Glutamic acid	G8415	1	G1001	1.5
Threonine	T8441	15	T8250	30
Phenylalanine	P5482	5	P1751	7.4
Proline	P5607	15	858919	147
Glutamine	G8540	5	G9003	29
Glycine	G7126	100	-	-
Serine	S4311	20	S4250	147
Arginine	A8094	35	A2646	0.3
Lysine	L5501	30	L8021	36.5
Alanine	A7469	85	A7377	73
Leucine	L8912	5	855448	7.4
Isoleucine	I7403	10	I7634	15
Valine	V0513	15	855987	19
Cysteine	C7352	45	30095	7
Asparagine	A4159	15	441597	19
Histidine	H6034	10	SC 255057	15
Aspartic acid	A7219	1	219096	1
Methionine	M5308	5	M9375	7.4
Tyrosine	T8566	0.4	855456	0.2
Tryptophan	T8941	2	T9753	0.7
**NON-CANONICAL CANDIDATES**	**Cat. No.**	**Conc (mM)**		
L-Norvaline	N7627	91.5		
D-Norvaline	851620	91.5		
L-2-Aminobutyric acid (L-AABA)	A1879	91.5		
D-2-Aminobutyric acid (D-AABA)	116122	91.5		
(S)-3-Aminobutyric acid ((S)-3-ABA)	757454	91.5		
4-Aminobutyric acid (4-ABA)	A2129	91.5		
β-Alanine	146064	91.5		
(R)-2-Hydroxybutyric acid	54917	91.5		
L-2-Hydroxyisovaleric acid	379093	91.5		
D-2-Hydroxyisovaleric acid	55452	91.5		
Sodium (R)-3-Hydroxy-isobutyrate	11161	91.5		
Sodium (S)-3-Hydroxy-isobutyrate	16842	91.5		
Sodium-3-Methyl-2-oxobutyrate	198994	91.5		
Butyric acid	B103500	91.5		
Isovaleric acid	129542	91.5		
**PURINES**	**Cat. No.**	**Conc (mM)**		
Hypoxanthine (Hx)	H9377	0.1		
Inosine (Ino)	I4125	0.1		
Adenosine (Ado)	A4036	0.1		
Xanthine (Xan)	X7375	0.1		
Xanthosine (Xao)	X0750	0.1		

Supplementary Table 3. Concentrations of all candidate germinants used in the screening experiments.

Figure	Term	Definition
	$OD_{(0)}, OD_{(t)}$	OD600 at time = t or 0 respectively
	$OD_{[+]}, OD_{[-]}$	OD600 in the presence [+] or absence [-] of the factor
	ΔOD_X	$OD_{(0)} - OD_{(t)}$ for condition X
	$\%\Delta OD_X$	$\frac{OD_{(0)}-OD_{(t)}}{OD_{(0)}} * 100\%$ for condition X
	$\overline{\Delta OD_X}$	$\frac{1}{N}\sum_{i=1}^{N} \Delta OD_{X,i}$ for condition X
	$\overline{\%\Delta OD_X}$	$\frac{1}{N}\sum_{i=1}^{N} \%\Delta OD_{X,i}$ for condition X
**Figs. 1b,2a,2d,3a**	ΔOD<br>(for each combination *c* among *N* combinations in one experiment)<br><br>*Note: this definition of ΔOD only applies to Figs. 1b, 2a, 2d, 3a*	$\frac{\Delta OD_C}{\max_n\{\Delta OD_1 \cdots \Delta OD_N\}}$
	Mean $\Delta OD_{[+]}$ - Mean $\Delta OD_{[-]}$<br>(for a single factor *f*)	$\overline{\Delta OD_{f,[+]}} - \overline{\Delta OD_{f,[-]}}$
**Figs. 1c,4a,5a,5d,6a,6b; Supplementary Figs. S2a,S2c,S2d**	Germination %	$\%\Delta OD_X$
**Figs. 2b,2e,3b**	Mean ΔOD<br>(for a single factor *f* when absent [-] or present [+])	$\overline{\Delta OD_{f,[+]}}$ or $\overline{\Delta OD_{f,[-]}}$
**Figs. 5c,6c**	Germination %<br>(for germinant *j* compared to D-valine at 91.5 mM)	$\frac{\%\Delta OD_j}{\%\Delta OD_{Dvaline}}$
**Supplementary Fig. S1**	Standardized effect<br>(for a single factor *f*)	$2 * \frac{\overline{\Delta OD_{f,[+]}} - \overline{\Delta OD_{f,[-]}}}{\sigma_M}$
**Supplementary Fig. S2a**	Mean $\%\Delta OD_{[+]}$ - Mean $\%\Delta OD_{[-]}$	$\overline{\%\Delta OD_{f,[+]}} - \overline{\%\Delta OD_{f,[-]}}$
**Supplementary Fig. S2b**	Mean %ΔOD<br>(for a single factor *f* when absent [-] or present [+])	$\overline{\%\Delta OD_{f,[+]}}$ or $\overline{\%\Delta OD_{f,[-]}}$
**Supplementary Fig. S4**	Relative OD	$\frac{OD_{(t)}}{OD_{(0)}}$

Supplementary Table 4. Summary table of formulae used to calculate the Y axes of graph figures. $OD_{(t)}$ refers to OD600 value at the end of the assay while $OD_{(0)}$ refers to the OD600 value at the start of the assay.

Figure	Graph description	Condition	Comparison with	n	Type of t-test	p value	Significance level
1c	Single factor screen L-amino acids	L-cysteine	L-valine	4	Unpaired	P<0.0001	****
		L-alanine	L-valine	4	Unpaired	P<0.0001	****
4a	L-amino acid D-amino acid inhibition	L-cysteine-D-cysteine	L-cysteine DIW	4	Unpaired	P<0.0001	****
		L-cysteine-D-alanine	L-cysteine DIW	4	Unpaired	P<0.0001	****
		L-cysteine-D-valine	L-cysteine DIW	4	Unpaired	0.7434	ns
		L-alanine-D-cysteine	L-alanine DIW	4	Unpaired	0.7851	ns
		L-alanine-D-alanine	L-alanine DIW	4	Unpaired	P<0.0001	****
		L-alanine-D-valine	L-alanine DIW	4	Unpaired	0.1281	ns
		L-valine-D-cysteine	L-valine DIW	4	Unpaired	P<0.0001	****
		L-valine-D-alanine	L-valine DIW	4	Unpaired	P<0.0001	****
		L-valine-D-valine	L-valine DIW	4	Unpaired	P<0.0001	****
5a	Single factor screen D-amino acids	D-valine	D-threonine	4	Unpaired	P<0.0001	****
5c	Dose response curve D-valine and other germinants	L-valine	D-valine	4	Unpaired	0.0001	****
		L-cysteine	D-valine	4	Unpaired	p<0.0001	****
		L-alanine	D-valine	4	Unpaired	0.0033	**
5d	D-valine interaction with other D- and L-amino acids	D-valine-D-cysteine	D-valine DIW	4	Unpaired	P<0.0001	****
		D-valine-D-alanine	D-valine DIW	4	Unpaired	P<0.0001	****
		D-valine-L-cysteine	D-valine DIW	4	Unpaired	0.2143	ns
		D-valine-L-alanine	D-valine DIW	4	Unpaired	0.1646	ns
		D-valine-L-valine	D-valine DIW	4	Unpaired	0.1575	ns
6a	Single factor screen D-valine analogs	(S)-3-ABA	4-ABA	4	Unpaired	0.0115	*
		L-AABA	4-ABA	4	Unpaired	P<0.0001	****
		D-AABA	4-ABA	4	Unpaired	0.0107	*
		L-norvaline	4-ABA	4	Unpaired	0.1083	ns
		D-norvaline	4-ABA	4	Unpaired	P<0.0001	****
		L-valine	4-ABA	4	Unpaired	0.0003	***
		D-valine	4-ABA	4	Unpaired	P<0.0001	****
6b	Valine analogs' interaction with other D-amino acids	L-AABA-D-cysteine	L-AABA DIW	4	Unpaired	0.0002	***
		L-AABA-D-alanine	L-AABA DIW	4	Unpaired	P<0.0001	****
		D-AABA-D-cysteine	D-AABA DIW	4	Unpaired	0.0002	***
		D-AABA-D-alanine	D-AABA DIW	4	Unpaired	P<0.0001	****
		L-norvaline-D-cysteine	L-norvaline DIW	4	Unpaired	0.1446	ns
		L-norvaline-D-alanine	L-norvaline DIW	4	Unpaired	0.0186	*
		D-norvaline-D-cysteine	D-norvaline DIW	4	Unpaired	P<0.0001	****
		D-norvaline-D-alanine	D-norvaline DIW	4	Unpaired	P<0.0001	****
		(S)-3-ABA-D-cysteine	(S)-3-ABA DIW	4	Unpaired	0.1513	ns
		(S)-3-ABA-D-alanine	(S)-3-ABA DIW	4	Unpaired	0.0044	**
6c	Dose response curve valine analogs	L-AABA	L-cysteine	4	Unpaired	0.1901	ns
		D-norvaline	L-cysteine	4	Unpaired	0.8813	ns
Supplementary Fig. S2c	Purines and L-cys single factor screen	Hx-L-cys 10 mM	Hx	4	Unpaired	P<0.0001	****
		Ado-L-cys 10 mM	Ado	4	Unpaired	P<0.0001	****
		Ino-L-cys 10 mM	Ino	4	Unpaired	P<0.0001	****
		Xao-L-cys 10 mM	Xao	4	Unpaired	P<0.0001	****
		Xan-L-cys 10 mM	Xan	4	Unpaired	0.0034	**
		Water-L-cys 10 mM	Water	4	Unpaired	0.0001	****
		Solvent-L-cys 10 mM	Solvent	4	Unpaired	P<0.0001	****
Supplementary Fig. S2d	L-cys-Hyp-Ino physiological concentration	DIW L-cys 1 mM	DIW-DIW	4	Unpaired	0.0754	ns
		Hx-L-cys 1 mM	Hx-DIW	4	Unpaired	0.0007	***
		Ino-L-cys 1 mM	Ino-DIW	4	Unpaired	0.0196	*
		Hx-Ino L-cys 1mM	Hx-Ino-DIW	4	Unpaired	0.0997	ns
		DIW L-cys 10 mM	DIW-DIW	4	Unpaired	P<0.0001	****
		Hx L-cys 10 mM	Hx-DIW	4	Unpaired	P<0.0001	****
		Ino L-cys 10 mM	Ino-DIW	4	Unpaired	P<0.0001	****
		Hx-Ino L-cys 10 mM	Hx-Ino -DIW	4	Unpaired	P<0.0001	****

Supplementary Table 5. Summary table of statistical analysis parameters for all non-combinatorial experiments in the study. Table shows the p values of column wise comparison of each condition with relevant controls for all single factor experiments in the study. All comparisons utilized the student's unpaired t-test where n refers to the number of biologically independent samples used for the tests. Significance levels were indicated using the following convention ****p≤0.0001, ***p≤0.001, **p≤0.01, *p≤0.05, ns p>0.5. ABA refers to aminobutyric acid.

C. novyi-NT gene query	Protein hit	Organism	E value	% Identity	Accession number
NT01CX_0562	Spore germination protein GerKC [Bacillus]	*Bacillus* spp.	3.00E-06	20.58	WP_000484123.1
NT01CX_0563	Spore germination protein GerIB	*B. cereus* 10876	4.00E-19	21.26	AAD03542.1
NT01CX_0564	GerA spore germination family protein	*B. cereus* 10876	1.00E-132	39.87	KFL74316.1
NT01CX_0189	Spore germination protein GerLC	*B. cereus* 10876	3.00E-10	22.17	AAK70463.1
NT01CX_0190	Spore germination protein GerIB	*B. cereus* 10876	2.00E-21	23.22	AAD03542.1
NT01CX_0191	Spore germination protein GerKA	*B. cereus* 10876	4.00E-96	34.23	EEK52439.1
NT01CX_0562	Ger(x)C family spore germination protein	*C. botulinum*	4.00E-106	39.09	WP_003356649.1
NT01CX_0563	Spore germination protein	*C. botulinum* A1 str CFSAN002368	4.00E-101	42.86	EPS52744.1
NT01CX_0564	Spore germination protein GerA	*C. botulinum* A1 str CFSAN002368	4.00E-74	35.47	EPS52609.1
NT01CX_0189	Germination protein GerC family	*C. botulinum* A1 str CFSAN002368	4.00E-15	66.67	EPS52611.1
NT01CX_0190	Spore germination protein	*C. botulinum* A1 str CFSAN002368	3.00E-23	25	EPS52744.1
NT01CX_0191	Spore germination protein GerA	*C. botulinum* A1 str CFSAN002368	2.00E-138	51.91	EPS52609.1

Supplementary Table 6. Prediction of putative germinant receptors in *C. novyi*-NT. Protein sequences from the *C.novyi*-NT genome predicted to be germination proteins were BLASTP queried against the genomes of *B. cereus* 10876 and *C. botulinum* strain A to identify analogous Ger family proteins.

	C. difficile	*C. frigidicarnis*	*C. perfringens* (Non foodborne)	*C. novyi*- NT	*C. perfringens* (foodborne)	*C.botulinum* (non-proteolytic)	*C.bifermentans*	*C.roseum*	*C.sordelli*	*C.sporogenes*	*C.botulinum* (proteolytic)	*C.butyricum*	*C. botulinum* group IV Type G	*C. tetani*	***Total***	*% of surveyed clostridia*
L-Alanine	✓	✓	✓	✓		✓	✓	✓	✓	✓	✓				**10**	71.4%
L-Cysteine	✓	✓	✓	✓	✓	✓						✓	✓		**8**	57.1%
L-Phenylalanine	✓						✓	✓	✓						**4**	28.6%
L-Serine		✓	✓		✓	✓									**4**	28.6%
L-Threonine		✓	✓		✓										**3**	21.4%
L-Norvaline	✓	✓		✓											**3**	21.4%
L-Arginine	✓						✓	✓	✓						**4**	28.6%
L-Valine		✓	✓	✓											**3**	21.4%
L-2-Aminobutyric acid	✓			✓											**2**	14.3%
Glycine	✓	✓													**2**	14.3%
L-Asparagine			✓		✓										**2**	14.3%
L-Methionine														✓	**1**	7.1%
L-Glutamine					✓										**1**	7.1%
**Total**	**7**	**7**	**6**	**5**	**5**	**3**	**3**	**3**	**3**	**1**	**1**	**1**	**1**	**1**		
% of surveyed L-amino acids	53.8%	53.8%	46.2%	38.5%	38.5%	23.1%	23.1%	23.1%	23.1%	7.7%	7.7%	7.7%	7.7%	7.7%		

Supplementary Table 7. Comparison of *C. novyi*-NT pro-germination L-amino acid factors with other Clostridia adapted from Bhattacharjee *et al* [45]. Green shaded boxes with ticks indicate pro-germination factors for corresponding Clostridia.

Figure	Model type	Factors	# of Factors	SD	R^2
1b, S1	Plackett-Burman	L-amino acids	20	0.123	92.1%
2a,b,c	Full factorial	L-amino acid germinants	5	0.256	74.14%
2d,e,f	Full factorial	L-cys and L-amino acid germination antagonists	5	0.086	93.07%
3a,b,c	Full factorial	Purines (in the presence of L-cys)	5	0.219	72.46%

Supplementary Table 8. Model fit parameters for the various DOE experimental designs performed in this study.

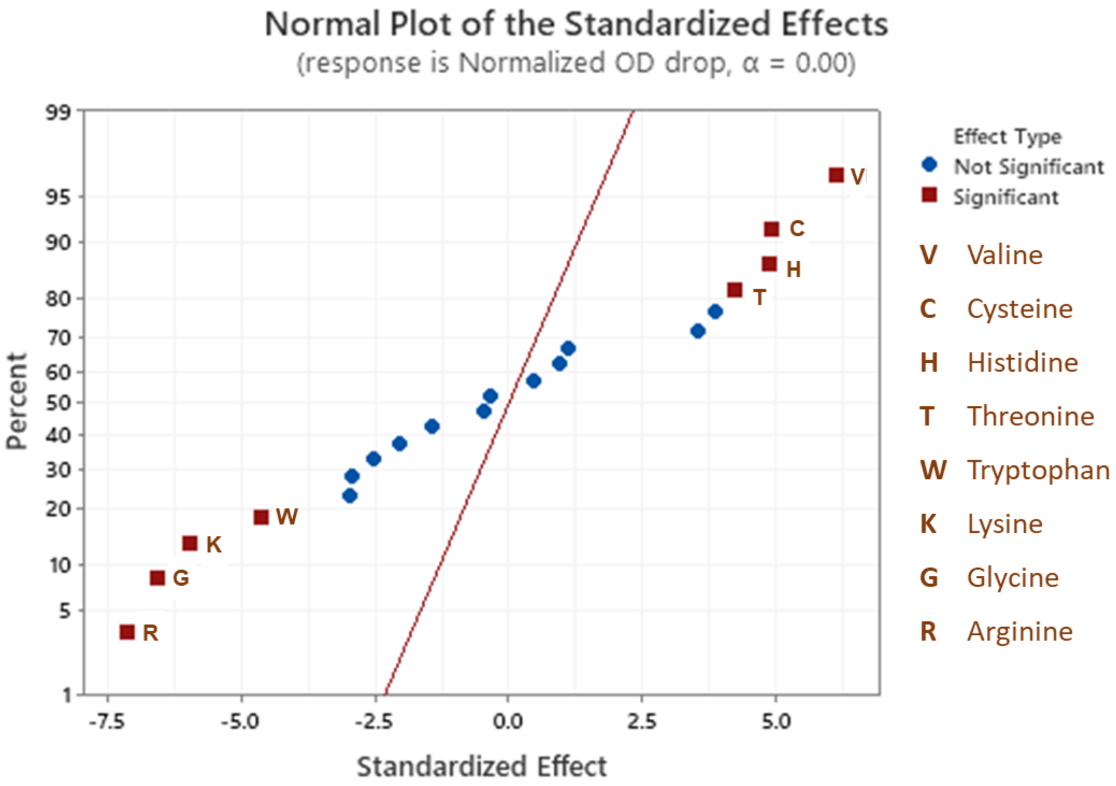

Supplementary Fig. S1. **Identification of significant pro-germination factors and antagonists.** 20 L-amino acids at concentrations in **Supplementary Table 3** were added in different combinations according to the Plackett-Burman (PB) design in **Supplementary Table 1**. The normal probability plot of the standardized effects as defined in **Supplementary Table 4** is shown for all 20 L-amino acid factors (α = 0.0005). Solid line represents line fit to a normal distribution. Significant factor effects are indicated by red squares while non-significant factor effects are indicated by blue circles.

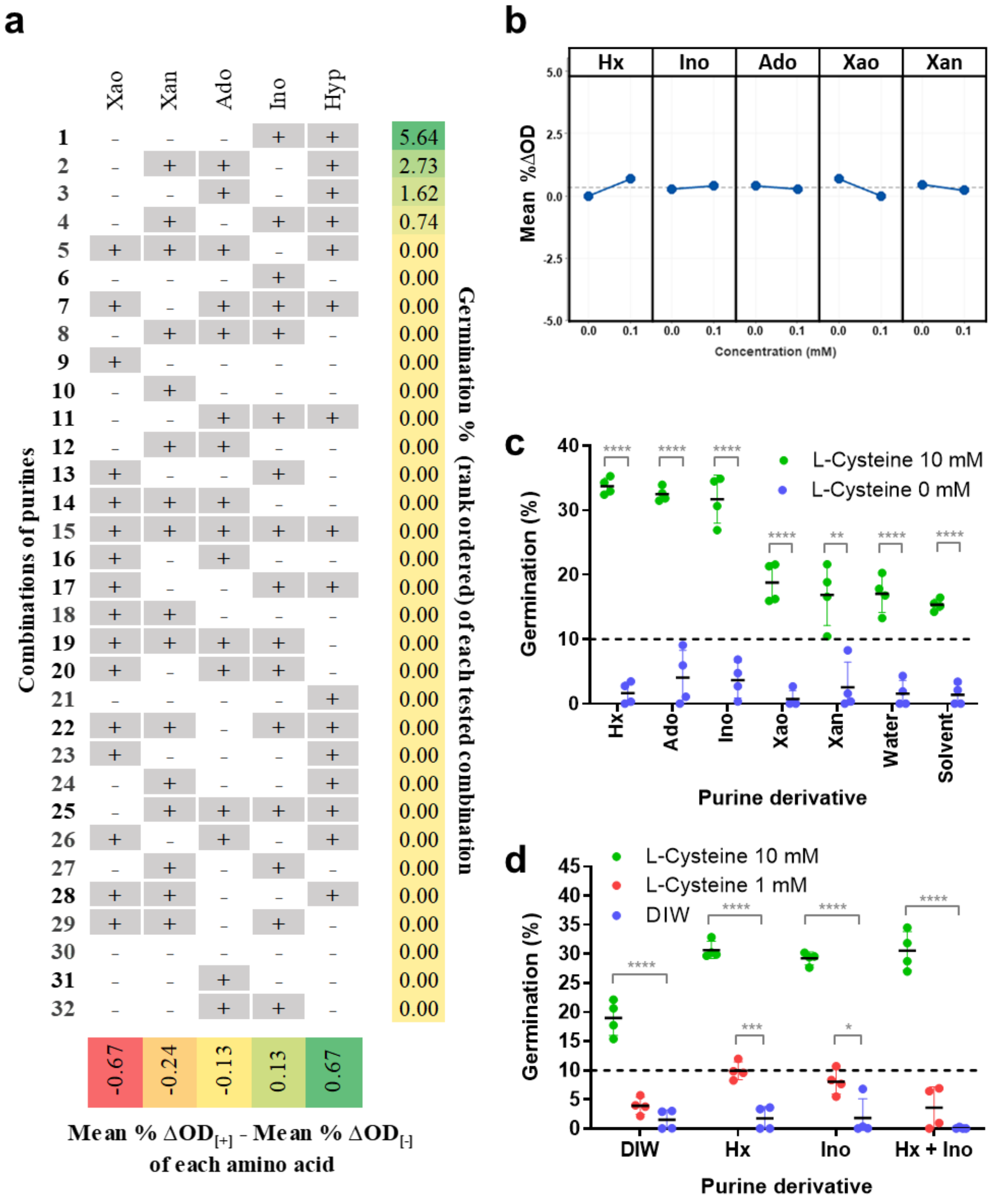

Supplementary Fig. S2. **Purine germinant and co-germinant screens. (a)** Figure depicts the different combinations of purines in the absence of main germinant L-cysteine according to the factorial design in **Supplementary Table 2**, sorted in decreasing order (from top to bottom) of average values of germination % as defined in **Supplementary Table 4**. Figure also shows the effect (bottom row) of each purine on the germination % arranged in increasing order from left to right. **(b)** Main interaction plot for full factorial design with purines (0.1 mM) in absence of L-cysteine (blue lines with circles, model α = 0.0005). **(c)** Single factor co-germinant screen in presence of L-cysteine (10 mM) and No L-cysteine; Purine concentration (0.1 mM). Solvent control refers to control with diluted NaOH used for dissolution of purines. Significant at ****$p \leq 0.0001$, ***$p \leq 0.001$, **$p \leq 0.01$ (unpaired t-test) when compared with no L-cysteine for each purine and control. **(d)** Germination in presence of L-cysteine (1 mM), hypoxanthine and inosine (0.1 mM) at physiological concentrations. Significant at ****$p \leq 0.0001$, ***$p \leq 0.001$, **$p \leq 0.01$, *$p \leq 0.05$, ns $p > 0.5$ when compared with DIW control without L-cysteine for each condition. All data were averaged from two independent experiments with two replicates each and depict mean response values for a, b and mean±SD, n=4 biologically independent samples for c, d. Green, red, blue circles indicate 10 mM, 1 mM and 0 mM L-cysteine respectively in c, d.

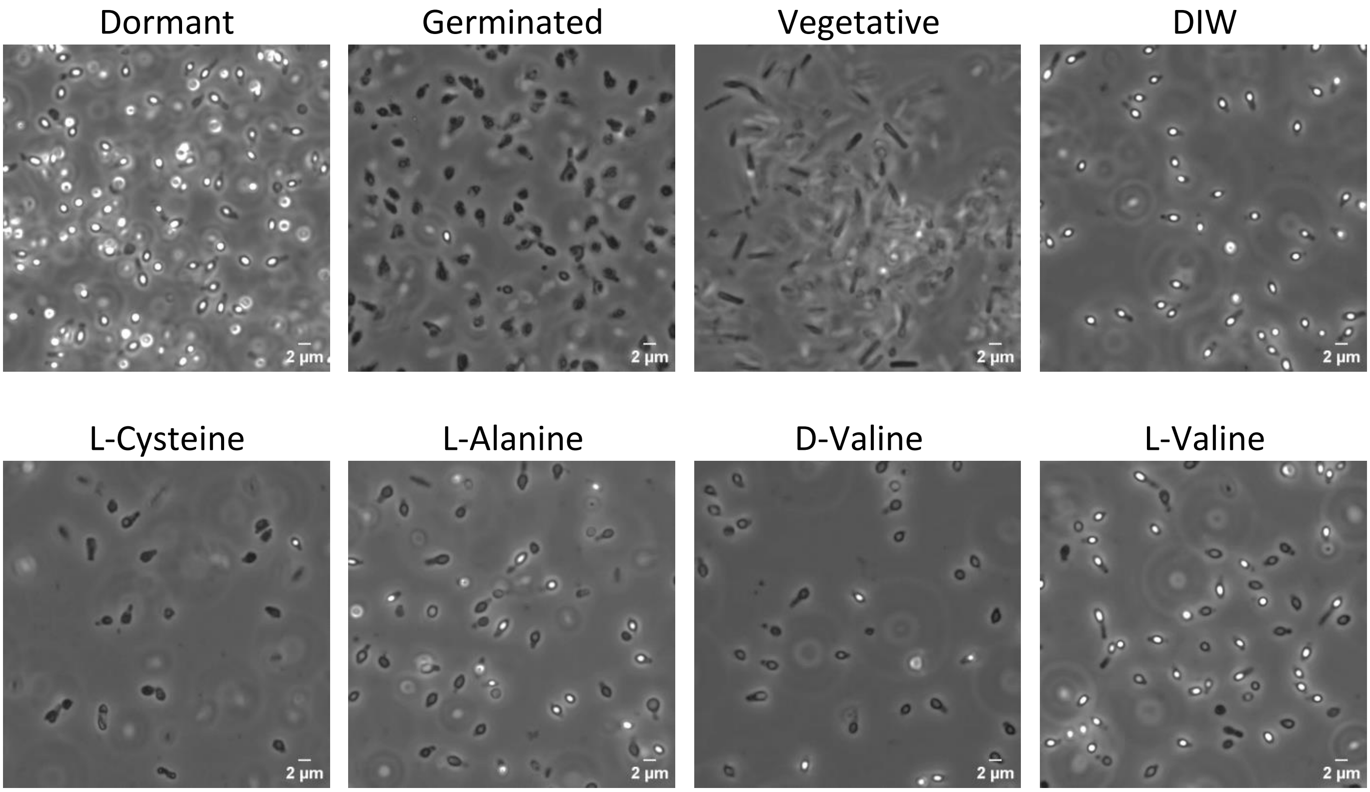

Supplementary Fig. S3. Phase contrast images of samples used for LC-MS analysis. Figure shows best representative phase contrast images of dormant, germinated and vegetative *C. novyi*-NT spore samples used for peptidoglycan extract analysis and spore samples used for germination with L-cysteine, L-alanine, L-valine, D-valine or DIW for the supernatant analysis. DIW refers to water control containing oxyrase. 1600X magnification, Scale bar 2 µm.

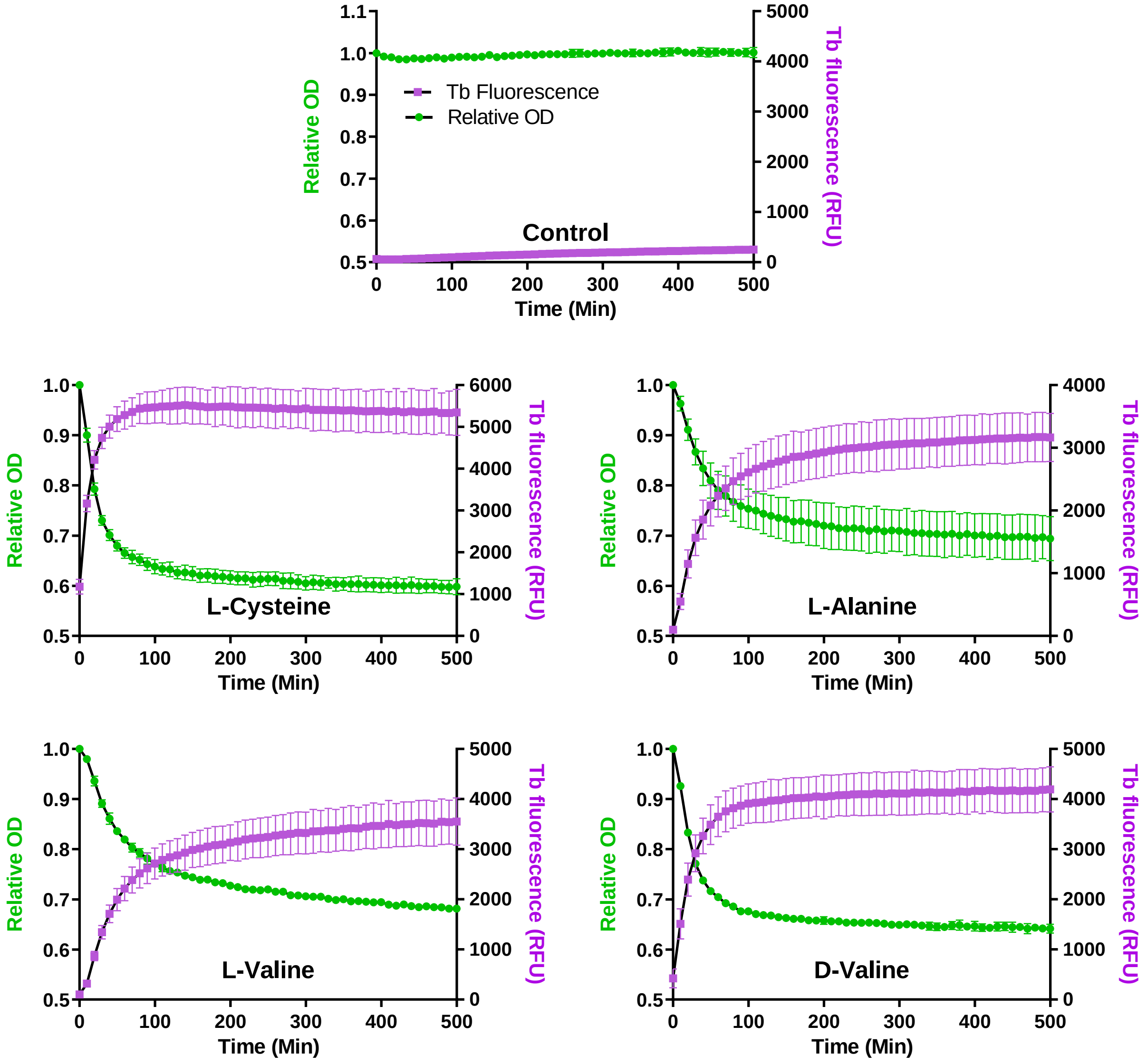

Supplementary Fig. S4. **D-Valine shows similar germination and DPA release kinetics as L-cysteine.** Spores were germinated with L-cysteine, L-alanine, L-valine, D-valine and water control, with an amino acid concentration of 93.5 mM in the presence of Terbium chloride (0.09 mM). All germination data depict mean ± SD, n=4 biologically independent samples. The OD600 relative to the initial starting OD600 (green circles) as defined in **Supplementary Table 4** is measured on the left Y-axis. The increase in Tb fluorescence over time at 545 nm (purple squares) is measured on the right Y-axis.

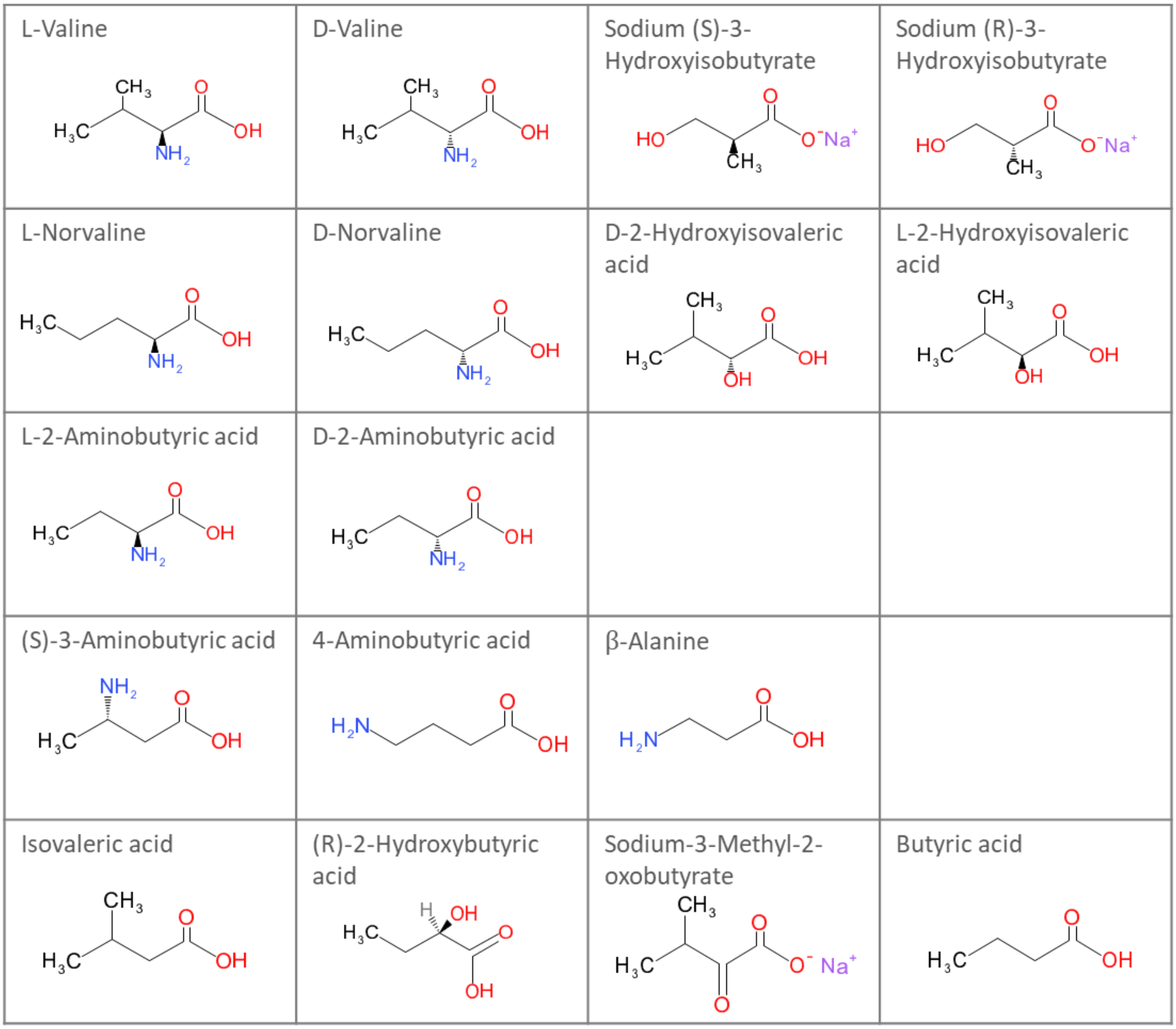

Supplementary Fig. S5. Chemical structures of all stereoisomeric valine analogs used in the study. Structures drawn using the Chem4Word plug-in in Microsoft word.

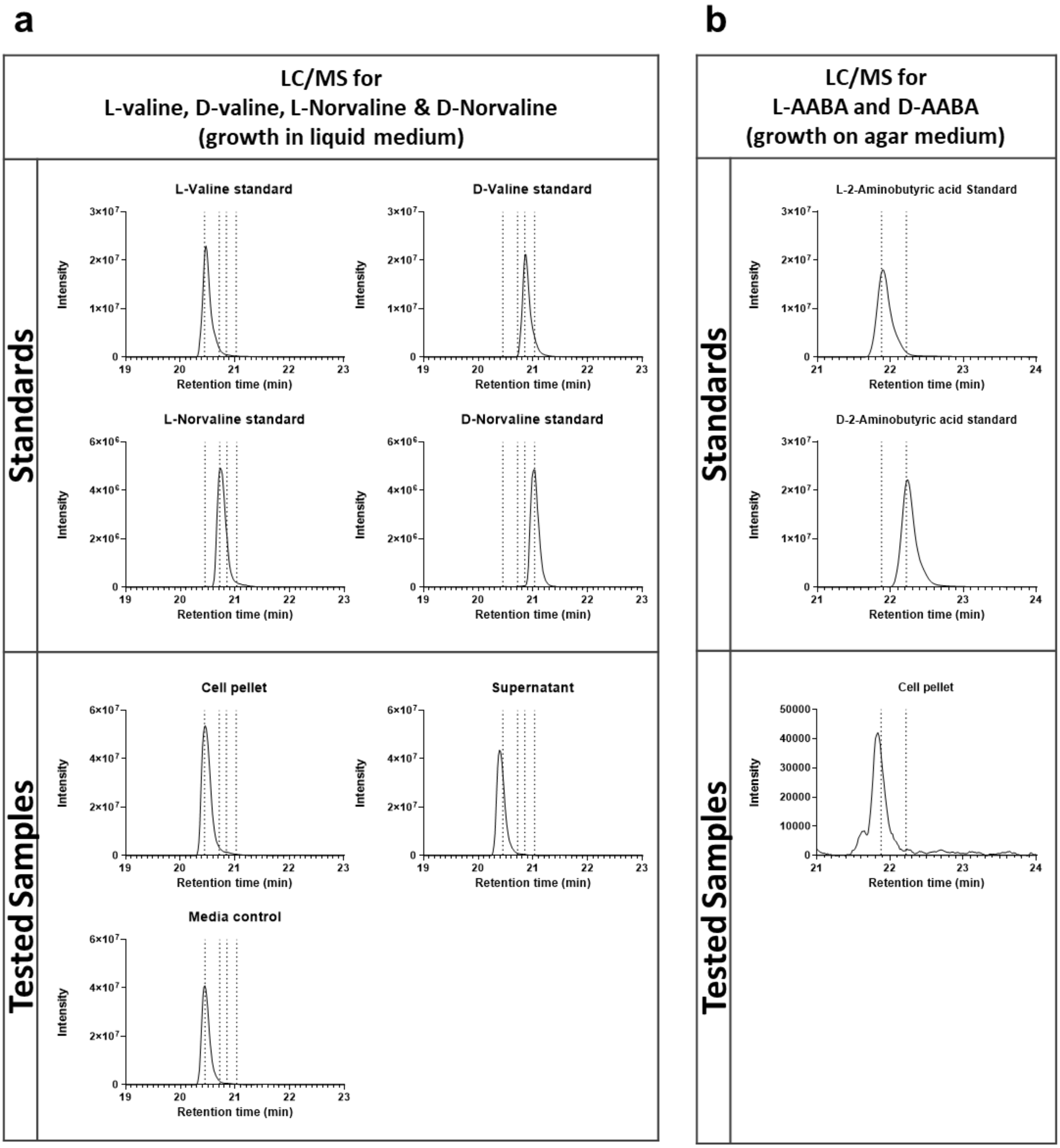

Supplementary Fig. S6. **L-2-aminobutyric acid is an intracellular metabolite produced in vegetative *C. novyi*-NT.** Overnight cultures of vegetative *C. novyi*-NT in BHI-FBS liquid medium (a) or agar plates (b) were harvested in 1X PBS and intracellular metabolites were extracted in 50% Acetonitrile after bead-beating. **(a)** LC-MS EIC spectra of derivatized Valine/Norvaline ion (379 m/z) for standards of L-valine, D-valine, L-norvaline, D-norvaline, and samples of Supernatant, Cell pellet and Media blank control. Data is best representative of water blank subtracted spectra from two independent experiments. Dotted lines represent the peak maxima for L-valine, D-valine, L-norvaline and D-valine at retention times of 20.45, 20.85, 20.72 and 21.03 min respectively. **(b)** LC-MS EIC spectra of derivatized 2-Aminobutyric acid ion (365 m/z) for Standards of L-2-aminobutyric acid, D-2-aminobutyric acid and samples of Cell pellet. Data represents media blank subtracted average spectra from two independent experiments. Spectra smoothened by using a moving average of six data points. Dotted lines represent the standard peaks for L-2-aminobutyric acid and D-2-aminobutyric acid at retention times of 21.88 and 22.22 min respectively.
