# Supplementary material for: A design of experiments screen reveals that Clostridium novyi-NT spore germinant sensing is stereoflexible for valine and its analogs
Source: Commun Biol. 2023 Jan 28;6:118. doi: 10.1038/s42003-023-04496-9 (PMC9884283; doi:10.1038/s42003-023-04496-9)
Supplement: Supplementary file 2 — Description of Additional Supplementary Data [file 42003_2023_4496_MOESM2_ESM.docx]

**Description of Additional Supplementary Files**

**File name:** Supplementary Data 1

**Description:** The source data behind graphs in the paper
