# Supplementary material for: A design of experiments screen reveals that Clostridium novyi-NT spore germinant sensing is stereoflexible for valine and its analogs
Source: Commun Biol. 2023 Jan 28;6:118. doi: 10.1038/s42003-023-04496-9 (PMC9884283; doi:10.1038/s42003-023-04496-9)
Supplement: Supplementary file 4 — Reporting Summary [file 42003_2023_4496_MOESM4_ESM.pdf]

nature portfolio

Corresponding author(s): Ian Cheong

Last updated by author(s): Oct 26, 2022

Reporting Summary

Nature Portfolio wishes to improve the reproducibility of the work that we publish. This form provides structure for consistency and transparency in reporting. For further information on Nature Portfolio policies, see our Editorial Policies and the Editorial Policy Checklist.

Statistics

For all statistical analyses, confirm that the following items are present in the figure legend, table legend, main text, or Methods section.

n/a	Confirmed	
☐	☒	The exact sample size (*n*) for each experimental group/condition, given as a discrete number and unit of measurement
☐	☒	A statement on whether measurements were taken from distinct samples or whether the same sample was measured repeatedly
☐	☒	The statistical test(s) used AND whether they are one- or two-sided<br>*Only common tests should be described solely by name; describe more complex techniques in the Methods section.*
☒	☐	A description of all covariates tested
☐	☒	A description of any assumptions or corrections, such as tests of normality and adjustment for multiple comparisons
☐	☒	A full description of the statistical parameters including central tendency (e.g. means) or other basic estimates (e.g. regression coefficient) AND variation (e.g. standard deviation) or associated estimates of uncertainty (e.g. confidence intervals)
☐	☒	For null hypothesis testing, the test statistic (e.g. *F*, *t*, *r*) with confidence intervals, effect sizes, degrees of freedom and *P* value noted<br>*Give P values as exact values whenever suitable.*
☒	☐	For Bayesian analysis, information on the choice of priors and Markov chain Monte Carlo settings
☐	☒	For hierarchical and complex designs, identification of the appropriate level for tests and full reporting of outcomes
☐	☒	Estimates of effect sizes (e.g. Cohen's *d*, Pearson's *r*), indicating how they were calculated

Our web collection on statistics for biologists contains articles on many of the points above.

Software and code

Policy information about availability of computer code

Data collection	Metamorph (Molecular devices, 7.10.3) was used for microscopic imaging. Spark Control Dashboard (Tecan, V2.2) was used for all the OD600 measurements.
Data analysis	MassHunter Qualitative navigator (Agilent, B.08.00) was used to analyze LC/MS EIC spectra. Minitab 20 was used for Design of Experiments analyses. Graphpad Prism was used for generating graphical plots and performing statistical analyses.

For manuscripts utilizing custom algorithms or software that are central to the research but not yet described in published literature, software must be made available to editors and reviewers. We strongly encourage code deposition in a community repository (e.g. GitHub). See the Nature Portfolio guidelines for submitting code & software for further information.

Data

Policy information about availability of data

All manuscripts must include a data availability statement. This statement should provide the following information, where applicable:

- Accession codes, unique identifiers, or web links for publicly available datasets
- A description of any restrictions on data availability
- For clinical datasets or third party data, please ensure that the statement adheres to our policy

All data generated or analysed during this study are included in this published article (and its supplementary information files).

Human research participants

Policy information about studies involving human research participants and Sex and Gender in Research.

Reporting on sex and gender	NA
Population characteristics	NA
Recruitment	NA
Ethics oversight	NA

Note that full information on the approval of the study protocol must also be provided in the manuscript.

Field-specific reporting

Please select the one below that is the best fit for your research. If you are not sure, read the appropriate sections before making your selection.

☒ Life sciences ☐ Behavioural & social sciences ☐ Ecological, evolutionary & environmental sciences

For a reference copy of the document with all sections, see nature.com/documents/nr-reporting-summary-flat.pdf

Life sciences study design

All studies must disclose on these points even when the disclosure is negative.

Sample size	More than 3.2x10^8 CFU/ml of spores were used for each germination experiment while spores of the order of 10^9 CFU/ml were used for the LC/MS experiments. This is far in excess of an amount which could cause error due to under-sampling.
Data exclusions	Fig. 1C - One biological replicate for L-asparagine was excluded for being a clear outlier. The criteria for outlier detection was pre-established as: [xi - median(xi)]/(median of all absolute deviations from the median) at a cutoff of 2.5. Including the outlier does not change the result of the experiment.
Replication	All attempts at replicating our findings were successful. DOE experiments were replicated twice with two embedded technical replicates. Single factor spore germination assays were performed with 4 biological replicates. The LC-MS and imaging data were best representative of two independent samples.
Randomization	Randomization was not relevant to this study.
Blinding	Blinding is not relevant when analyzing bacteria or bacterial spores. Experimental replicates and embedded technical replicates ensured reproducibility.

Reporting for specific materials, systems and methods

We require information from authors about some types of materials, experimental systems and methods used in many studies. Here, indicate whether each material, system or method listed is relevant to your study. If you are not sure if a list item applies to your research, read the appropriate section before selecting a response.

Materials & experimental systems

n/a	Involved in the study	
☒	☐	Antibodies
☒	☐	Eukaryotic cell lines
☒	☐	Palaeontology and archaeology
☒	☐	Animals and other organisms
☒	☐	Clinical data
☒	☐	Dual use research of concern

Methods

n/a	Involved in the study	
☒	☐	ChIP-seq
☒	☐	Flow cytometry
☒	☐	MRI-based neuroimaging
